# Novel role of the class II α-isoform of phosphatidylinositol 3-kinase in hepatocellular carcinoma: suppressing metastasis by targeting Wnt/β-catenin signaling via inhibiting phosphorylation of LRP6

**DOI:** 10.1186/s12935-026-04368-2

**Published:** 2026-06-22

**Authors:** Jing Chen, Li-Xin Ren, Yong Ma, Wen-Hua Cheng, Ting Liu

**Affiliations:** 1 Department of breast surgery, Shanxi Cancer Hospital, Taiyuan, Shanxi China; 2 Department of Hepatobiliary, Pancreatic and Gastrointestinal Surgery, Shanxi Cancer Hospital, Taiyuan, Shanxi China; 3 Department of Thoracic surgery, Shanxi Cancer Hospital, Taiyuan, Shanxi China; 4 Department of Gastroenterology, Shanxi Cancer Hospital, Taiyuan, Shanxi China; 5 Department of General Surgery, Shanxi Hospital Affiliated to Cancer Hospital, Shanxi Cancer Hospital, Chinese Academy of Medical Sciences, Cancer Hospital Affiliated to Shanxi Medical University, Taiyuan, 030032 China

**Keywords:** Hepatocellular carcinoma, PIK3C2A, Prognosis, Metastasis, Wnt/β-catenin signaling

## Abstract

Hepatocellular carcinoma (HCC) is one of the most lethal cancers worldwide because of metastasis. The class II α-isoform of phosphatidylinositol 3-kinase (PIK3C2A) was found to be a regulator in proliferation and migration of endothelial cells, but its role in cancer metastasis has less been evaluated. In this study we find it functions as a tumor suppressor. PIK3C2A was frequently down-regulated in human HCC cells and tissues. Low PIK3C2A expression correlated with elevated LRP6 phosphorylation, β-catenin nuclear accumulation, and Wnt/β-catenin hyperactivation in clinical HCC tissues. Low PIK3C2A predicted poor overall and disease-free survival and aggressive clinicopathological features. Loss of PIK3C2A significantly promoted HCC cells invasion and migration in vitro, and promoted tumor growth and metastasis in vivo. And these changed phenotypes by PIK3C2A knockdown in HCC cells could be rescued by exogenous expression of PIK3C2A. Mechanism studies demonstrated loss of PIK3C2A activated Wnt/β-catenin signaling dramatically. Further investigations showed loss of PIK3C2A gave rise to a decrease in phosphatidylinositol (3,4) bisphosphate (PI(3,4)P_2_) and an increase in phosphatidylinositol (4,5) bisphosphate (PI(4,5)P_2_), therefore promoting phosphorylation of low-density lipoprotein receptor-related protein 6 (LRP6). Moreover, the activated Wnt/β-catenin signaling and the significantly increased invasion and migration cells by loss of PIK3C2A were also markedly suppressed in various levels respectively by inhibiting LRP6 phosphorylation in diverse levels. Inhibiting LRP6 phosphorylation abolished PIK3C2A-mediated effects. Taken together, our results indicate PIK3C2A suppresses HCC metastasis by targeting Wnt/β-catenin signaling via inhibiting phosphorylation of LRP6, and can serve as a prognostic biomarker and therapeutic target for HCC.

**Supplementary Information:**

The online version contains supplementary material available at 10.1186/s12935-026-04368-2.

## Introduction

Hepatocellular carcinoma (HCC) in men is the fifth estimated new cases and second leading cause of cancer-related deaths worldwide. In 2012, about 782,500 new liver cancer cases and 745,500 deaths occur worldwide, with China accounting for half of the total number of cases and deaths [1]. However, curative therapy only suits a minority of patients with relative small tumor burdens and well compensated liver function [2]. What’s worse, more than 70% of them will undergo tumor recurrence in five years after hepatic resection [3]. Therefore, illuminating the mechanisms of metastasis and recurrence in HCC is of great importance to identify a new precise therapy strategy.

The phosphatidylinositol 3-kinase (PI3K) family participates in diverse cellular processes, including cell proliferation, migration, and vesicular trafficking [4]. Mammalian PI3Ks are categorized into three subclasses (Class I, II and III), the Class I PI3Ks are well-characterized oncogenic molecules, which activate downstream AKT cascade by catalyzing PIP2 conversion to PIP3, thereby facilitating malignant progression, immune escape and chemotherapy resistance in various tumors [5]. In contrast, the class II α-isoform of phosphatidylinositol 3-kinase (PIK3C2A) is mainly localized in cell membrane, the Golgi apparatus and clathrin-coated vesicles [6]. Existing studies have confirmed that PIK3C2A participates in physiological angiogenesis and vascular barrier maintenance, and regulates the proliferation and migration of vascular endothelial cells [7, 8]. Notably, a previous study reported that PIK3C2A mRNA functions as a miR-124 sponge to positively regulate CD151 expression and enhance the migration and invasion abilities of HCC cells, highlighting its pro-metastatic role in HCC [9]. Compared with the clearly defined pro-tumor effects of Class I PI3Ks, the biological functions and clinical significance of Class II PI3Ks remain poorly elucidated, especially in the context of HCC tumorigenesis and metastasis [10].

Low-density lipoprotein receptor-related protein 6 (LRP6) acts as an essential co-receptor for canonical Wnt/β-catenin signaling, cooperating with Wnt ligands and Frizzled receptors to trigger downstream signal transduction [11]. In HCC, aberrant LRP6 overexpression and hyperphosphorylation are strongly linked to aggressive clinicopathological characteristics, including vascular invasion, intrahepatic spread and advanced tumor stage, and serve as independent risk factors for poor prognosis [12]. Constitutive LRP6 activation leads to persistent Wnt/β-catenin cascade hyperactivation, thereby facilitating HCC cell proliferation, invasion and metastasis [13]. Clinically, excessive Wnt signaling activation correlates with high postoperative recurrence and inferior survival, highlighting its potential as a viable therapeutic target for HCC intervention [14].

Although the oncogenic mechanisms of Class I PI3Ks and Wnt/β-catenin signaling have been extensively investigated in HCC, the crosstalk between PIK3C2A members and Wnt cascades remains largely unreported. In particular, whether PIK3C2A regulates LRP6 phosphorylation to mediate Wnt pathway activation and HCC metastasis still lacks experimental verification, representing a critical research gap in this field. In this study, we find that low PIK3C2A expression level is closely correlated with poor prognosis of HCC patients, and loss of PIK3C2A promotes HCC metastasis. Mechanism studies show PIK3C2A suppresses Wnt/β-catenin signaling via direct inhibition on the phosphorylation of LRP6 in HCC.

## Materials and methods

### Patients and tissue specimens

A total of 130 pairs of randomly selected HCCs and adjacent non-tumorous liver tissues (ANLTs) from consecutive patients who have received hepatic resection for HCC at Department of Surgery, Cancer Hospital Affiliated to Shanxi Medical University (Taiyuan, China) from January 2005 to December 2010, were enrolled into training cohort. And 71 pairs of randomly selected HCCs and ANLTs from consecutive HCC patients received hepatic resection at Department of Abdominal Surgical Oncology, Taiyuan Cancer Hospital (Taiyuan, China) from January 2004 to December 2009, were enrolled into validation cohort (Fig. S1). The samples were snap-frozen in liquid nitrogen and stored at -80℃ for later RNA extraction or formalin-fixed and paraffin embedded for imunohistochemistry. The study protocol followed the Ethical Guidelines of the 1975 Declaration of Helsinki, revised in 2000. Prior informed consent was obtained from all patients. Researches on human materials were approved by the Ethics Committee of the Affiliated Cancer Hospital of Shanxi Medical University and the Ethics Committee of Taiyuan Cancer Hospital. Diagnosis of HCC was confirmed by two independent histopathologists. The follow-up period was defined as the interval between the date of operation and that of the patient’s death or the last follow-up. Deaths from other causes were treated as censored cases. Surveillance for recurrence and metastasis were conducted by clinical examination, serial AFP level, and ultrasonography or computed tomography (CT) scan every 3 months in the first 2 years, and twice a year in the next years. Disease-free survival was defined as the length of time after liver resection during which a patient survived without sign of HCC. The complete clinical and pathological features of these patients were collected and stored in our database by a researcher fellow. All research protocols strictly complied with REMARK guidelines for reporting prognostic biomarkers in cancer [15].

### Multi-pathway reporter array

A Cignal Finder 10-Pathway Reporter Array (QIAGEN) was adopted for the pathway analysis. Cells were infected with knockdown lentivirus as well as its negative control (NC) lentivirus. Relative firefly luciferase activity was calculated and normalized to the constitutively expressed Renilla luciferase. The assay was performed as protocol provided by the kit.

### Lipid extraction and protein lipid overlay assay

Cellular lipids were extracted from HCC cells (5 × 10^6^ cells / sample) after different treatments. Media were firstly removed by gentle aspiration and immediately add 5 mL ice cold 0.5 M TCA into each flask. Scrape them down and Centrifuge at 1500 RPM for 5 min. Discard the supernatant. Add 3 mL 5% TCA/ 1 mM EDTA to the pellet. Vortex to re-suspend. Centrifuge at 1500 RPM for 5 min. Discard the supernatant. Add 2.25 mL MeOH : CHCl_3_ : 12 M HCl (80:40:1) and vortex 4 times over 15 min at room temperature. Centrifuge at 1500 RPM for 5 min and obtain the supernatant. Add 0.75 mL of CHCl_3_ and 1.35 mL of 0.1 M HCl. Vortex. Centrifuge at 1500 RPM for 5 min to separate organic and aqueous phases. Collect organic (lower) phase and dry in a vacuum dryer.

For protein lipid overlay assay [16], the dried lipid samples were firstly reconstituted with CHCl_3_: MeOH: H_2_O (1: 2: 0.8). And then 2µL lipid sample was spotted onto a nitrocellulose membrane and dried. Membranes were blocked in PBS containing 3% BSA at room temperature for 1 h. For the detection of phosphatidylinositol (3,4) bisphosphate (PI(3,4)P_2_), the membrane was incubated with mouse anti-PI(3,4)P_2_ antibody (Echlon Biosciences) and then anti-mouse IgG-HRP (Sigma). For the detection of phosphatidylinositol (4,5) bisphosphate (PI(4,5)P_2_), the membrane was incubated sequentially with PI(4,5)P_2_ Grip™ (Echlon Biosciences), mouse anti-GST antibody (Sigma) and anti-mouse IgG-HRP (Sigma). The complex on the membrane was eventually detected with enhanced chemiluminescence regents (Thermo Scientific). Images of the spots were analyzed by densitometry analysis software.

### HCC mouse model and in vivo imaging study

The HCC model in mice was constructed as described. Briefly, 5 × 10^6^ cells were injected subcutaneously into the left upper flank regions of nude mouse (3–4 weeks of age, male, BALB/c). One month later, the subcutaneous tumor tissues were removed and diced into 1 mm^3^ cubes, which were then implanted into the liver of nude mouse (6 in each group). For the in vivo imaging study (IVIS), different group of cells were labeled with firefly luciferase under the help of Shanghai Sciencelight Biology Science & Technology Co., Ltd (Shanghai, China). The in vivo tumor formation and metastases were imaged by bioluminescence. D-luciferin (Sciencelight) at 150 mg/kg was injected intraperitoneally into the mice, and the bioluminescence was detected and captured under IVIS lumina II (Xenogen). The condition of tumors in each group was detected every week according to the protocol of IVIS. After 6 weeks, all mice were sacrificed, and their organs were harvested for IVIS. Liver orthotopic tumors of each group were measured and tumor volume was estimated by the following formula: (Length ×Width^2^)/2 [17]. Then their lungs were fixed with phosphate-buffered neutral formalin. Serial sections were subjected to histopathological analysis by hematoxylin and eosin (H&E) staining, and the pulmonary metastatic foci was confirmed and recorded by specialized pathologists. All experimental procedures were approved by the Animal Ethics Committee of the Shanxi Medical University.

### Other materials and methods

Details for cell lines and cell culture, RT-PCR, qRT-PCR, Western blot, Immunohistochemistry, Immunofluorescence, transfection, and in vitro cell behavior assays, were described in the Supplementary Materials and Methods.

### Statistical analysis

All data were analyzed using the statistical software SPSS 17 for Windows (SPSS Inc.). Comparisons were made for the differences in clinicopathological features. The correlation between PIK3C2A expression and clinicopathological parameters was analyzed by Spearman rank-correlation analysis. Kaplan-Meier method constructed survival curves and evaluated the difference of these groups by using the log-rank test. The Cox proportional hazard regression model was used to identify the risk factors that were independently associated with overall survival and disease-free survival. Only factor which was *P* < 0.05 in univariate analysis could be analyzed in multivariate Analysis. Continuous data in this study were presented as mean ± standard deviation (SD) from at least three independent experiments. The differences between groups were analyzed by Student’s *t* test when only two groups were compared or by one-way analysis of variance (ANOVA) when more than two groups were compared. Categorical data were analyzed with χ^2^ test or Fisher’s exact test. A two-tailed *P* < 0.05 was considered statistically significant.

## Results

### PIK3C2A is frequently down-regulated in human HCC cells and tissues

**Fig. 1:**
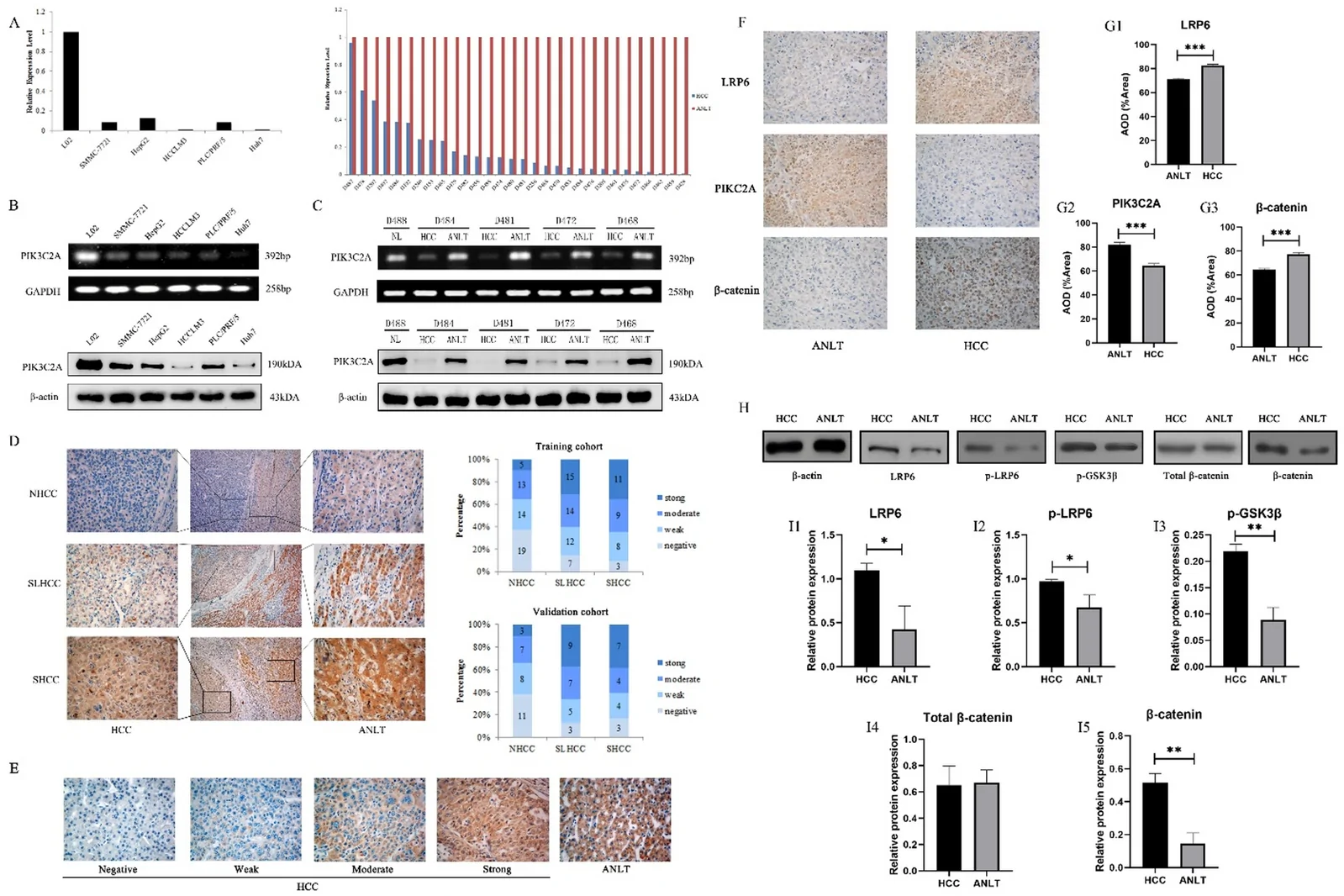
PIK3C2A is frequently down-regulated in human HCC cells and tissues. (**A**) PIK3C2A is down-regulated in human HCC cells and 30 paired tissues analyzed by qRT-PCR. (**B**) PIK3C2A is down-regulated in human HCC cells analyzed by RT-PCR and Western Blot. (**C**) PIK3C2A is down-regulated in human HCC tissues analyzed by RT-PCR and Western Blot. (**D**) Representative immunohistochemistry (IHC) images of PIK3C2A expression in HCC and ANLT from NHCC, SLHCC and SHCC. Magnifications: ×100, ×400. Bar graph shows statistics for staining intensity of NHCC, SLHCC and SHCC in training and validation cohorts. (**E**) Representative IHC images of different staining intensity of PIK3C2A in HCC and representative IHC images of PIK3C2A expression in ANLT. Magnifications: ×100, ×400. (**F**) Representative IHC staining of LRP6, PIK3C2A, and β-catenin in paired HCC and ANLT samples.(**G**) Quantification of IHC staining intensity for LRP6, PIK3C2A, and β-catenin. (**H**) Western blot analysis of LRP6, p-LRP6, p-GSK3β, total β-catenin, and nuclear β-catenin in paired HCC and ANLT tissues. (I) Quantitative analysis of Western blot results for LRP6, p-LRP6, p-GSK3β, total β-catenin, and nuclear β-catenin. *P < 0.05, **P < 0.01, ***P < 0.001 vs. ANLT

To study the expression of PIK3C2A in human HCC cells and tissues, we performed real-time PCR, RT-PCR and Western Blot. Firstly, PIK3C2A mRNA was detected by real-time PCR in cells and 30 paired tissues (SHCC (Small HCC, single nodule ≤ 5 cm) = 8; SLHCC (Solitary Large HCC [18], single nodule > 5 cm) = 8; NHCC (nodular HCC, node number ≥ 2) = 14). Compared with immortalized human normal liver cells L02, PIK3C2A mRNA was down-regulated in HCC cells (Fig. 1A). Consistently, in contrast with their matched adjacent non-tumorous liver tissues (ANLTs), PIK3C2A mRNA was also low-expressed in HCC tissues (Fig. 1A). These results were then corroborated by RT-PCR and Western Blot performed in HCC cells and 4 paired tissues (Fig. 1B, C). Particularly, we simultaneously detect the expression level of PIK3C2A in normal liver tissue (D488) by RT-PCR and Western Blot. And in comparison with normal liver tissue, PIK3C2A in HCCs were also in a low level (Fig. 1C).

Moreover, PIK3C2A expression in HCC from two cohorts of 130 and 71 HCC patients was further analyzed by immunohistochemistry (IHC). It suggested that PIK3C2A protein was frequently down-regulated in HCC (Fig. 1D). And PIK3C2A expressed low in 64.7% (33/51) and 65.5% (19/29) of NHCC, which is of high-metastasis potential and poor prognosis after hepatectomy, as compared with 39.6% (19/48) and 33.3% (8/24) of SLHCC and 35.5% (11/31) and 38.9% (7/18) of SHCC, both of which exhibits a low invasive and metastatic potential and a good outcome after resection [18] (Fig. 1D). Consistently, PIK3C2A expression in high-metastasis potential cell lines, such as HCCLM3 is also lower than that in low-metastasis potential cell lines PLC/PRF/5, SMMC7721 and HepG2 (Fig. 1A, B). These data indicated PIK3C2A was down-regulated in HCC and it may be correlated with HCC invasion and metastasis.

Furthermore, we validated the downstream signaling in clinical HCC tissues. IHC and Western blot analyses showed that LRP6 and phosphorylated LRP6 were significantly upregulated in HCC tissues compared with ANLTs (Fig. 1G1, I1, I2). β-catenin nuclear accumulation was markedly increased in HCC, while total β-catenin showed no obvious difference (Fig. 1G3, I4, I5). In addition, phosphorylated GSK3β was also elevated in HCC tissues (Fig. 1I3). These clinical results confirmed that low PIK3C2A expression was closely associated with enhanced LRP6 phosphorylation and activation of the Wnt/β-catenin pathway in human HCC.

### Low expression of PIK3C2A in HCC tissues is associated with aggressive clinicopathological features and shorter postoperative survival rate

**Fig. 2:**
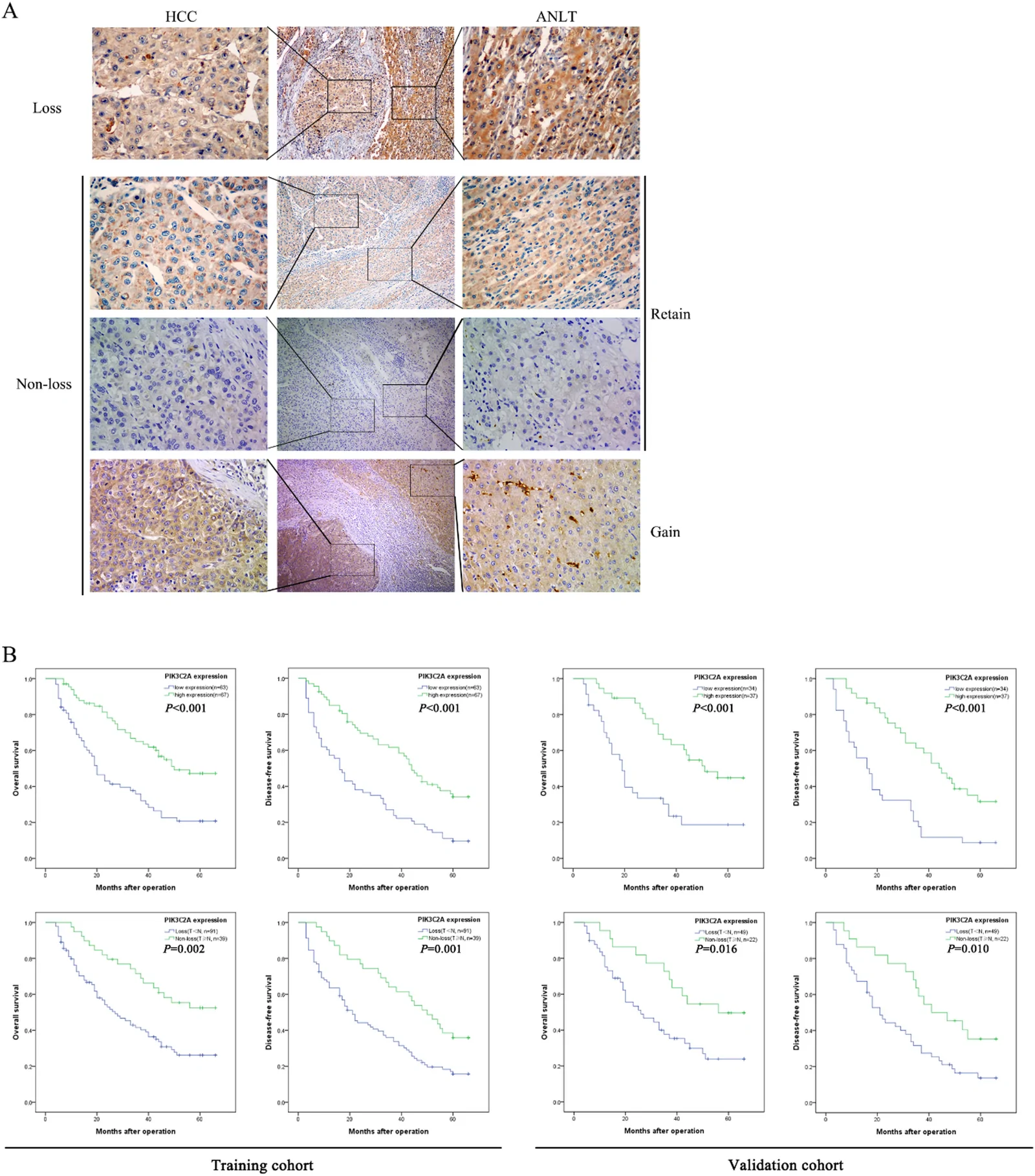
Low expression of PIK3C2A in HCC tissues is associated with aggressive clinicopathological features and shorter postoperative survival rate. (**A**) Representatives IHC images of PIK3C2A loss / non-loss (retain/gain) cases. Magnifications: ×100, ×400. (**B**) The overall survival (OS) and disease-free survival (DFS) of HCC patients with low / high PIK3C2A expression and PIK3C2A loss / non-loss in training cohort and validation cohort. Survival curve was calculated with the log-rank test and *P* value was shown in each panel respectively

To clarify the clinical relevance of PIK3C2A expression in HCC, two independent cohorts (training cohort and validation cohort) were adopted (Supplementary Table 1) and patients were dichotomized according to high (strong or moderate) or low (weak or negative) expression of PIK3C2A (Fig. S1A). In training cohort, it showed that the expression level of PIK3C2A was significantly associated with the following clinicopathological features: tumor nodule number, capsular formation, venous invasion, subtype of HCC, TNM stage and BCLC stage (all *P* < 0.05, Table 1). We next analyzed the relationship between PIK3C2A expression and the patients’ prognosis. The univariate and multivariate analysis revealed that low PIK3C2A expression was an independent risk factor for both overall survival (OS) and disease-free survival (DFS) of HCC patients after liver resection (Supplementary Tables 2–3). Furthermore, HCC patients in low PIK3C2A expression group had lower overall survival (OS) (1-, 3-, 5-year OS: 68.9%, 35.8%, 20.7% vs. 89.3%, 65.1%, 47.3%, *P* < 0.001) and disease-free survival (DFS) (1-, 3-, 5-year DFS: 57.1%, 27.0%, 9.5% vs. 85.0%, 61.5%, 34.1%, *P* < 0.001) than patients with high expression group (Fig. 2B). PIK3C2A was an independent prognosis factor and its low expression associated with poor survival of HCC patients were further validated in validation cohort (Fig. 2B, Supplementary Tables 4–6).

**Table 1:** The correlations of PIK3C2A with clinicopathological features of HCC

Clinicopathologic Variable	n	PIK3C2A		P
		Low expression	High expression	
Gender				
Female	21	10	11	
Male	109	53	56	0.933
Age(year)				
≤60	87	43	44	
＞60	43	20	23	0.754
AFP				
<20ng/ml	61	28	33	
≥20ng/ml	69	35	34	0.583
HBsAg				
Negative	21	13	8	
Positive	109	50	59	0.178
Liver cirrhosis				
Absence	49	26	23	
Presence	81	37	44	0.414
Tumor size(cm)				
≤5	57	25	32	
＞5	73	38	35	0.354
Tumor nodule number				
Solitary	79	30	49	
Multiple(≥2)	51	33	18	0.003
Capsular formation				
Presence	64	20	44	
Absence	66	43	23	<0.001
Edmondson-Steiner grade				
Ⅰ-Ⅱ	77	34	43	
Ⅲ-Ⅳ	53	29	24	0.236
Venous invasion				
Absence	74	21	53	
Presence	56	42	14	<0.001
Subtype of HCC				
SHCC	31	11	20	
SLHCC	48	19	29	
NHCC	51	33	18	0.011
TNM stage				
Ⅰ	55	12	43	
Ⅱ	31	18	13	
Ⅲ	44	33	11	<0.001
BCLC stage				
0-A	37	18	19	
B	66	23	43	
C	27	22	5	<0.001
Liver function				
Child-Pugh A	91	40	51	
Child-Pugh B	39	23	16	0.116
AFP, alpha-fetoprotein; HBsAg, hepatitis B surface antigen; TNM, tumor node metastasis; BCLC, Barcelona Clinic Liver Cancer				

As patients’ ANLTs showed varied intensity of PIK3C2A, which may be due to their different genetic background, we further assessed the significance of PIK3C2A loss and gain in tumor compared with corresponding non-tumorous liver background. We firstly divided patients of training cohort into three groups: PIK3C2A loss group, whose tumor showed lower expression than ANLT (*n* = 91); PIK3C2A retain group, whose tumor and ANLT had equivalent expression (*n* = 37); and PIK3C2A gain group, whose tumor showed higher expression than ANLT (*n* = 2). Since there were only two patients in PIK3C2A gain group, we then merged PIK3C2A retain group and gain group into PIK3C2A non-loss group (*n* = 39). And thus patients were dichotomized into PIK3C2A loss group (T < N) and non-loss group (T ≥ N) (Fig. 2A, S1B). The result showed that HCC patients in PIK3C2A loss group had lower OS (1-, 3-, 5-year OS: 72.6%, 41.6%, 26.2% vs. 94.9%, 71.5%, 52.5%, *P* = 0.002) and DFS (1-, 3-, 5-year DFS: 63.6%, 36.1%, 15.7% vs. 89.7%, 64.1%, 35.9%, *P* = 0.001) than patients in PIK3C2A non-loss group (Fig. 2B). The prognostic value of PIK3C2A loss / non-loss were further verified in validation cohort (Fig. 2B).

Taken together, these data fully demonstrated that PIK3C2A was closely correlated with poor survival and could be used as a novel independent prognosis biomarker for HCC patients after hepatic resection.

### Loss of PIK3C2A promotes invasion and migration of HCC cells in vitro and tumor metastasis in vivo

**Fig. 3:**
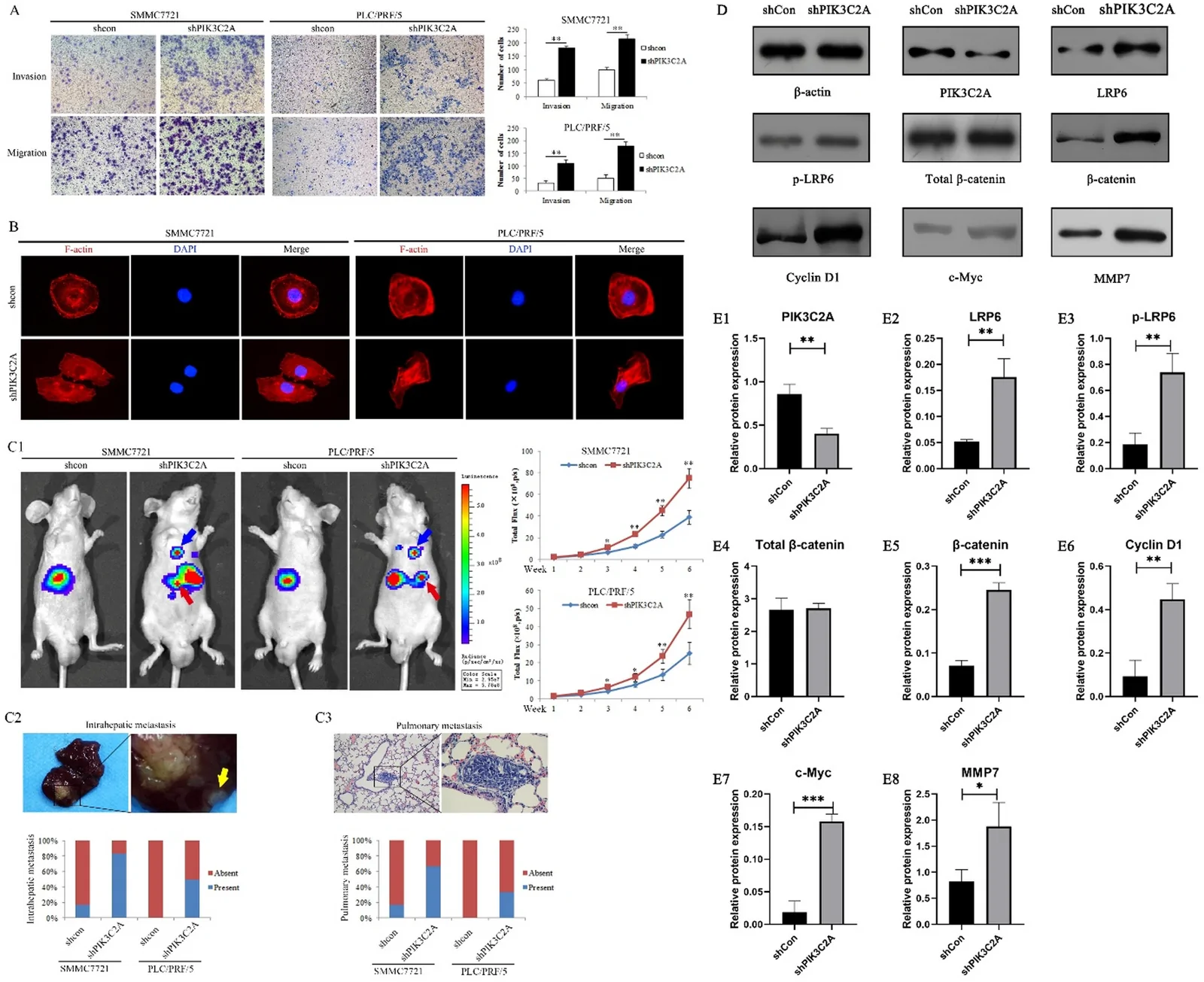
Loss of PIK3C2A promotes invasion and migration of HCC cells in vitro and tumor metastasis in vivo. (**A**) Effects of PIK3C2A silencing on cell invasion and migration using Transwell invasion / migration assay. Representative images of invasive or migrated cells are shown (left panel). Magnification: ×100. Results represented the mean ± SD in triplicate using bar graph (right panel). **P<0.01. (**B**) Effects of PIK3C2A silencing on cytoskeleton analyzed by F-actin fluorescent staining. Original magnification: ×400. (**C**) Effects of PIK3C2A silencing on in vivo tumor growth and metastasis. (C1) Representative bioluminescent imaging of the different groups is shown at 6 weeks after orthotopic implantation (left panel). Blue arrow indicates lung metastatic nodule and red arrow indicates intrahepatic metastasis. The growth curve showed the total flux of orthotopic tumors in each group every week detected by IVIS indicating the growth rate of tumors (right panel). *P<0.05, **P<0.01. (C2) Representative pictures of intrahepatic metastasis (upper panel). Yellow arrow indicates intrahepatic metastasis. Bar graph shows statistics for the percentage of mice with or without metastatic nodules in the livers (lower panel). (C3) Representative pictures of pulmonary metastasis using Hematoxylin and eosin (H&E) staining (upper panel). Magnifications: ×100, ×400. Bar graph shows statistics for the percentage of mice with or without metastatic nodules in the lungs (lower panel). (**D**) Representative Western blot images showing the expression of PIK3C2A, LRP6, p-LRP6, total and nuclear β-catenin, Cyclin D1, c-Myc, and MMP7 in shCon and shPIK3C2A groups. β-actin was used as the loading control. (**E**) Quantitative analysis of the Western blot results. Data are presented as mean ± SD; *P < 0.05, **P < 0.01, ***P < 0.001 vs. shCon

To explore the exact function of PIK3C2A in HCC, SMMC7721and PLC/PRF/5, both of which were of high PIK3C2A expression and low-metastasis potential cell lines, were transfected with lentiviral construct targeting coding sequences of PIK3C2A along with negative control. Two short hairpin RNAs (shRNA-1, shRNA-2) were used and their silencing efficiency were measured by real-time PCR (Fig. S2A). And shRNA-2 was the most effective one and was further identified by Western Blot and chosen for further study (Fig. S2B). We firstly used wound-healing assay to evaluate the migration capacity and results showed cells with PIK3C2A knocked down had a markedly faster wound closure rate (Fig. S2C). Next we performed Transwell invasion / migration assay. And invasion and migration of both SMMC7721 and PLC/PRF/5 cells were significantly and dramatically increased after knocking down PIK3C2A (Fig. 3A). Moreover, we also adopted F-actin fluorescent staining for cytoskeleton analysis. Compared with control cells exhibited a cobblestone shape and shrinkable F-actin fibers, treated cells presented an elongated cellular morphology and stretched F-actin fibers, which better suited cell migration (Fig. 3B). These in vitro data indicates loss of PIK3C2A promotes HCC cells invasion and migration. Furthermore, we also explored its function in proliferation of HCC cells. Colony formation assay showed a significant but slight increase in the number of colonies after knocking down PIK3C2A (Fig. S2D). Similar results were also gained in cell proliferation assay (Fig. S2E). And thus it suggests the function of PIK3C2A in proliferation of HCC cells is in a minor position.

Furthermore, these results were corroborated by in vivo assay. We performed IVIS to monitor the status and metastasis burden of mouse bearing xenografts and their expression of PIK3C2A in tumors of different groups were verified by Western Blot (Fig. S3A). After knocking down PIK3C2A, the growth rate of orthotopic tumors derived from shPIK3C2A cells was significantly but also slightly accelerated and their size were larger than that from shcon cells (Fig. 3C1, S3B). And the intrahepatic and pulmonary metastasis rate in mice with tumors derived from shPIK3C2A cells was significantly higher than that in mice with tumors derived from shcon cells (Fig. 3C, S3C), indicating loss of PIK3C2A mainly promotes invasion and metastasis of HCC and plays a minor role in accelerating proliferation.

Furthermore, Western blot analysis of mouse pulmonary metastatic lesions revealed that PIK3C2A was significantly downregulated in the shPIK3C2A group compared with the shcon group (Fig. 3E1). Meanwhile, LRP6 and p-LRP6 were markedly upregulated (Fig. 3E2, E3), nuclear β-catenin was increased (Fig. 3E5), and the downstream Wnt target genes c-Myc, Cyclin D1, and MMP7 were all elevated (Fig. 3E6, E7, E8), while total β-catenin showed no obvious change (Fig. 3E4). These in vivo data further confirmed that loss of PIK3C2A promotes HCC distant metastasis through hyperactivation of the LRP6/β-catenin signaling pathway.

### PIK3C2A knockdown phenotype in HCC cells can be rescued by exogenous expression of PIK3C2A

**Fig. 4:**
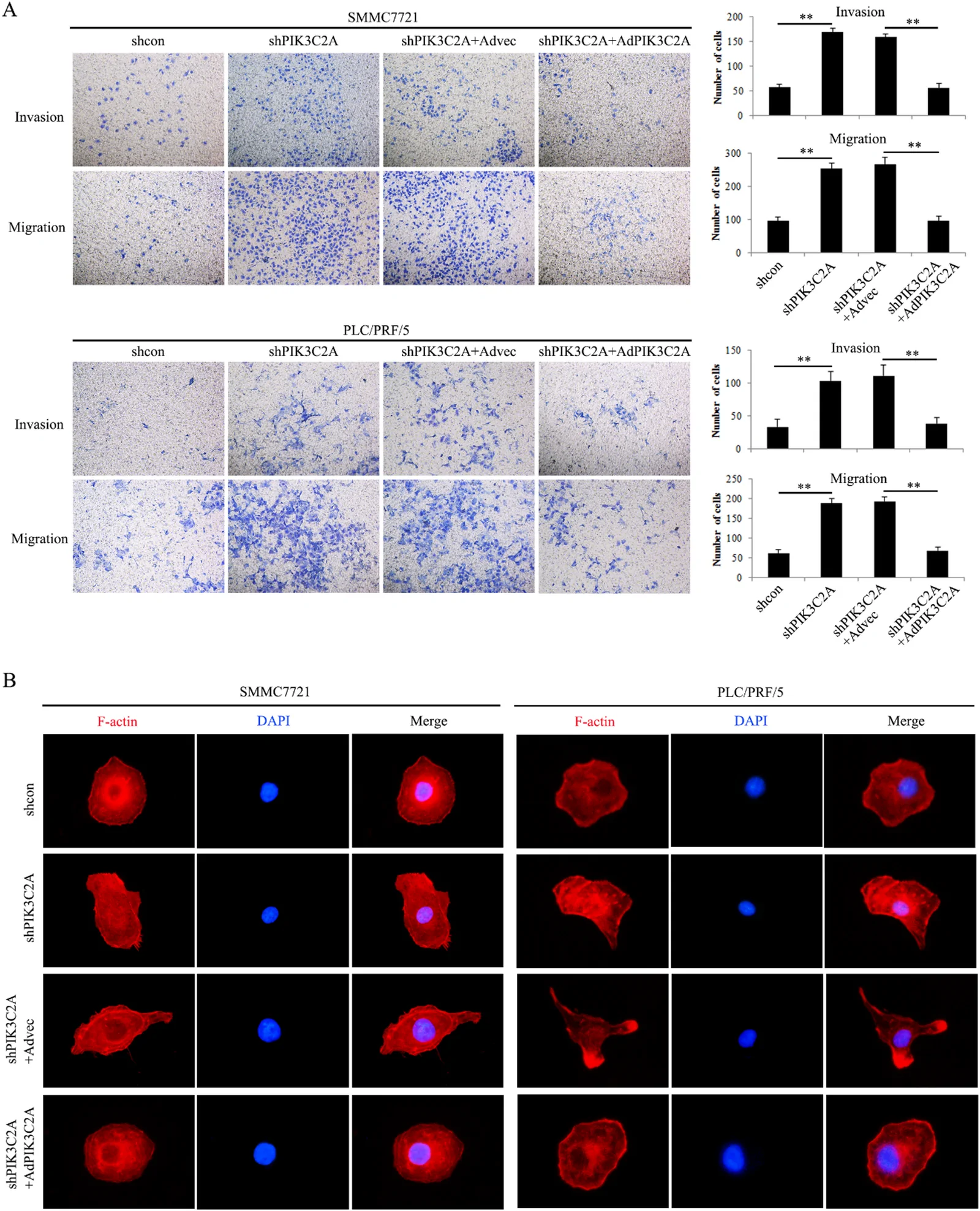
PIK3C2A knockdown phenotype in HCC cells can be rescued by exogenous expression of PIK3C2A. (**A**) Effects of PIK3C2A silencing and rescue on cell invasion and migration using Transwell invasion / migration assay. Representative images of invasive or migrated cells are shown (left panel). Magnification: ×100. Results represented the mean ± SD in triplicate using bar graph (right panel). **P<0.01. (**B**) Effects of PIK3C2A silencing and rescue on cytoskeleton analyzed by F-actin fluorescent staining. Original magnification: ×400

To fully demonstrate the role of PIK3C2A in HCC metastasis and exclude a bare possibility of off-target effects, we performed rescue experiment by using adenovirus containing mutated ORF nucleotide sequence of PIK3C2A, which was insensitive to selected shRNA targeting PIK3C2A but also expressed the same PIK3C2A protein. The sustaining time of PIK3C2A expressed in shPIK3C2A cells by adenovirus transfection was assessed (Fig. S4A) and it indicated the expression of PIK3C2A stabilized in 48 h and sustained about 10 days. That means all of the in vitro cell behavior assays except colony formation assay can be performed after the first-time transfection. And thus we added adenovirus in day 8 when performing colony formation assay. The rescue of PIK3C2A expression in shPIK3C2A cells was also verified by Western Blot (Fig. S4B). We then performed Transwell invasion / migration assay. The increased invasion and migration induced by loss of PIK3C2A were significantly decreased after overexpression of PIK3C2A in shPIK3C2A cells (Fig. 4A). And consistently in cytoskeleton analysis, elongated cellular morphology and stretched F-actin fibers of shPIK3C2A cells changed back to cobblestone shape and shrinkable F-actin fibers after PIK3C2A overexpression (Fig. 4B). Moreover, the slightly and significantly increased proliferation ability in shPIK3C2A cells was markedly inhibited after rescuing the expression of PIK3C2A (Fig. S4C, D). All these results suggested PIK3C2A knockdown phenotype in HCC cells can be rescued by exogenous expression of PIK3C2A, and it further corroborated the role of PIK3C2A in suppressing HCC metastasis.

### Loss of PIK3C2A activates Wnt/β-catenin signaling and abrogates its direct inhibition on LRP6 phosphorylation

**Fig. 5:**
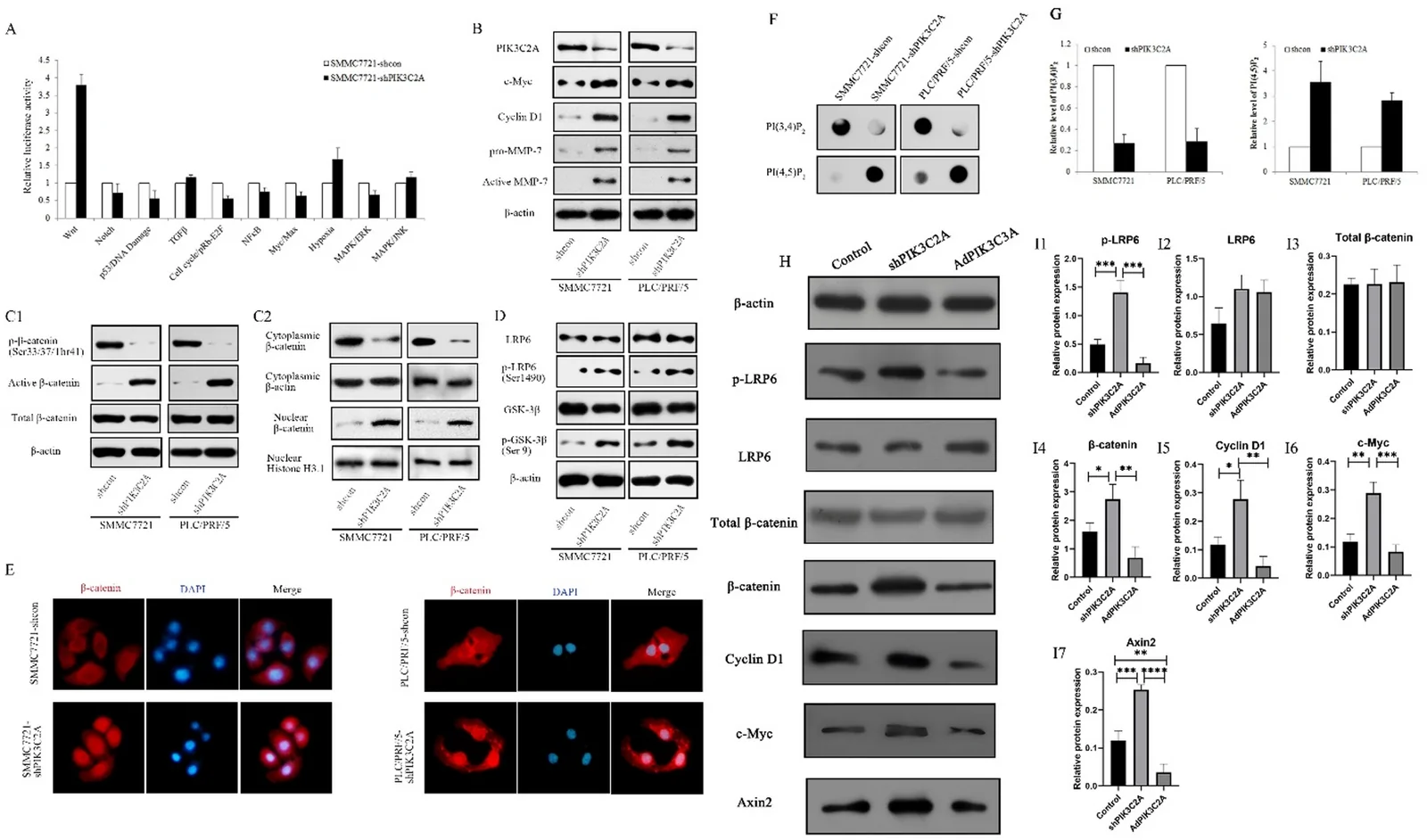
Loss of PIK3C2A activates Wnt/β-catenin signaling and abrogates its direct inhibition on LRP6 phosphorylation. (**A**) 10-Pathway Reporter Array showed the signaling change in PIK3C2A-interfered cells. (**B**) Protein levels of c-Myc, Cyclin D1 and MMP7 detected by Western Blot were shown in indicated cells. (**C**) Protein levels of phosphorylated-β-catenin, active β-catenin and total β-catenin detected by Western Blot were shown in indicated cells. (**D**) Protein levels of cytoplasmic and nuclear β-catenin detected by Western Blot were shown in indicated cells. (**E**) Protein levels of normal and phosphorylated form of LRP6 and GSK-3β detected by Western Blot were shown in indicated cells. (**F**) Immunofluorescence staining of β-catenin in indicated cells. Original magnification: ×100. (**G**) Representative results of protein lipid overlay assay of the PI(3,4)P_2_ and PI(4,5)P_2_ (left panel). Bar graph shows the relative level of PI(3,4)P_2_ and PI(4,5)P_2_ in shPIK3C2A cells compared with shcon cells (right panel). (**H**) Representative Western blot images of p-LRP6, LRP6, total β-catenin, nuclear β-catenin, Cyclin D1, c-Myc, and Axin2 in Control, shPIK3C2A, and AdPIK3C2A groups. β-actin was used as the loading control. (I) Quantitative analysis of the Western blot results. Data are presented as mean ± SD; *P < 0.05, **P < 0.01, ***P < 0.001

To systemically screen out the potential signaling manipulated by PIK3C2A, a Cignal Finder Cancer 10-Pathway Reporter Array was adopted [14]. And it indicated Wnt/β-catenin signaling was dramatically activated after knocking down PIK3C2A, and the luciferase activity of shPIK3C2A cells was 3.80 times that of shcon cells (Fig. 5A). We next performed Western Blot to justify the result. We firstly detected c-Myc, Cyclin D1, and matrix metalloproteinase 7 (MMP7), which were downstream proteins in Wnt/β-catenin signaling. Notably, they were all increased after knocking down PIK3C2A, especially the active form of MMP7 (Fig. 5B). As activating β-catenin and its nuclear localization was the mark of activating Wnt/β-catenin signaling [19], we then detected its phosphorylated and active (dephosphorylated) form, the total expression, and their expression in cytoplasm and nucleus respectively. We found loss of PIK3C2A inhibited β-catenin phosphorylation and increased its expression in nucleus, and the total expression was not changed (Fig. 5C, D). To find out in which way β-catenin were activated and thus nucleus localized, we continued to measure normal and phosphorylated form of glycogen synthase kinase 3β (GSK-3β) and low-density lipoprotein receptor-related protein 6 (LRP6), which were upstream protein of β-catenin in Wnt/β-catenin signaling. Results showed loss of PIK3C2A promoted the phosphorylation of GSK-3β and LRP6 (Fig. 5E). In addition, we performed immunofluorescence for cells to further corroborate the nuclear localization of β-catenin. Consistently, after knocking down PIK3C2A, the β-catenin in nucleus was increased evidently (Fig. 5F). And now all of these results indicated loss of PIK3C2A activated Wnt/β-catenin signaling and it might be through promoting LRP6 phosphorylation. We then tried to clarify how loss of PIK3C2A promoted LRP6 phosphorylation, and if it was a direct function.

We reviewed related literatures. Several reports [4, 6, 20] have suggested phosphatidylinositol (PI) and phosphatidylinositol 4-phosphate (PI(4)P) are preferred substrates of PIK3C2A, therefore generating phosphatidylinositol 3-phosphate (PI(3)P) and phosphatidylinositol (3,4) bisphosphate (PI(3,4)P_2_). And on the other hand, it is reported that Dishevelled (Dvl/Dsh), which relays Wnt signals from receptors to downstream effectors, can stimulate the formation of phosphatidylinositol (4,5) bisphosphate (PI(4,5)P_2_) by sequential actions of phosphatidylinositol 4-kinase type II (PI4KIIa) on PI and phosphatidylinositol-4-phosphate 5-kinase type I (PIP5KI) on PI(4)P [21–23]. And importantly, the resultant PI(4,5)P_2_ is required for Wnt-induced clustering and phosphorylation of LRP6 [21, 23]. Therefore, we hypothesized under the function of PIK3C2A, the generation of PI(3,4)P_2_ increased, and thus generating PI(4,5)P_2_ was competitively suppressed, which caused LRP6 phosphorylation was inhibited. We hence adopted protein lipid overlay assay to detect PI(3,4)P_2_ and PI(4,5)P_2_. Results demonstrated loss of PIK3C2A gave rise to a decrease in PI(3,4)P_2_ and an increase in PI(4,5)P_2_ (Fig. 5G), which indicating that loss of PIK3C2A can activate Wnt/β-catenin signaling and abrogate its direct inhibition on LRP6 phosphorylation.

Furthermore, consistent results were obtained in HepG2 cells. PIK3C2A knockdown markedly elevated p-LRP6 levels, promoted β-catenin nuclear accumulation, and upregulated the Wnt target genes c-Myc, Cyclin D1, and Axin2 (Fig. 5H and I). In contrast, overexpression of PIK3C2A exerted the opposite effects, reducing phosphorylated LRP6, retaining β-catenin in the cytoplasm, and downregulating the expression of c-Myc, Cyclin D1, and Axin2 (Fig. 5H and I). These data further verified that PIK3C2A negatively regulates the Wnt/β-catenin pathway in a dose‑dependent manner across multiple HCC cell lines.

### Inhibiting phosphorylation of LRP6 by LRP6 knockdown mimics impact of PIK3C2A on cell invasion and migration

**Fig. 6:**
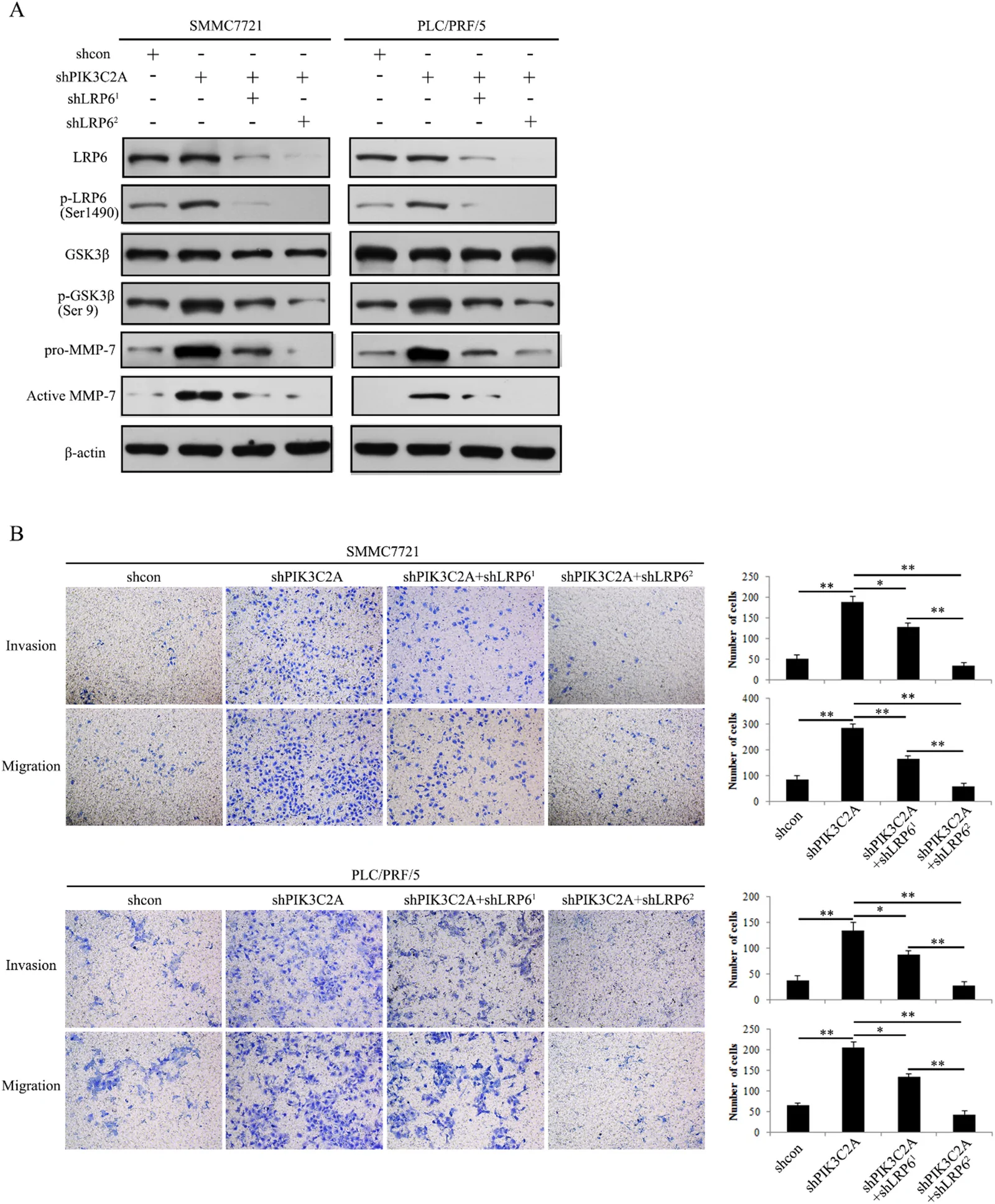
Inhibiting phosphorylation of LRP6 by LRP6 knockdown mimics impact of PIK3C2A on cell invasion and migration. (**A**) Protein levels of LRP6, p-LRP6, GSK-3β, p-GSK-3β and MMP7 detected by Western Blot were shown in indicated cells. shLRP6^1^ and shLRP6^2^ were the sequence of shLRP6-3 and shLRP6-1, which caused significant decrease of about 50% and 85% respectively in the expression of LRP6 mRNA validated by qRT-PCR. (**B**) Transwell invasion / migration assay were conducted in indicated cells to explore effects of inhibiting phosphorylation of LRP6 in different levels if can mimic the role of PIK3C2A on cell invasion and migration using. Representative images of invasive or migrated cells are shown (left panel). Magnification: ×100. Results represented the mean ± SD in triplicate using bar graph (right panel). *P<0.05, **P<0.01. shLRP6^1^ and shLRP6^2^ were the sequence of shLRP6-3 and shLRP6-1, which caused significant decrease of about 50% and 85% respectively in the expression of LRP6 mRNA validated by qRT-PCR

Having demonstrated PIK3C2A can directly inhibit LRP6 phosphorylation, we wanted to further clarify whether the inhibition effect of PIK3C2A on cell invasion and migration was through suppressing LRP6 phosphorylation. We adopted four LRP6 knockdown lentiviruses, which contained four different shRNA targeting LRP6 respectively, and measured silencing efficiency of each shRNA by qRT-PCR. Then shLRP6-1 and shLRP6-3, which caused significant decrease of about 85% and 50% respectively (Fig. S5), were chosen for the following research, to explore the role of different levels of LRP6 phosphorylation in cell invasion and migration. Firstly, LRP6, phosphorylated-LRP6, GSK-3β, phosphorylated-GSK-3β, and MMP7, which were all principal proteins of Wnt/β-catenin signaling, were detected by Western Blot. Notably, while total GSK-3β protein remained stable in PLC/PRF/5 cells, a mild reduction was observed in SMMC7721 cells after LRP6 knockdown (Fig. 6A). This cell-specific phenomenon likely reflects endogenous regulatory crosstalk within the Wnt pathway, in which reduced LRP6 signaling may subtly modulate GSK-3β protein stability in certain HCC cellular backgrounds. Importantly, this mild alteration did not affect the consistent trend of phosphorylated-GSK-3β and downstream MMP7 expression. Knocking down LRP6 by the two chosen shRNA caused different decreased levels of LRP6 and phosphorylated-LRP6, and accordingly, the increased expression of phosphorylated-GSK-3β and MMP7 by loss of PIK3C2A was also inhibited in different levels respectively (Fig. 6A). Furthermore, we adopted Transwell invasion / migration assay to corroborate it. Consistently, the significantly increased invasion and migration cells by loss of PIK3C2A were also markedly decreased in various levels respectively by inhibiting LRP6 phosphorylation in diverse levels (Fig. 6B). These data demonstrated the role of PIK3C2A in cell invasion and migration can be mimicked by inhibiting LRP6 phosphorylation. And thus we concluded that PIK3C2A suppressed HCC metastasis by targeting Wnt/β-catenin signaling via direct inhibition on phosphorylation of LRP6.

## Discussion

PI3Ks are a family capable of phosphorylating the 3’-position of the inositol ring of phosphoinositides. They are responsible for coordinating a diverse range of cell functions including proliferation, cell survival, cytoskeletal organization, vesicular trafficking and cell migration [24]. PIK3C2A, which is a member of PI3Ks, is incompletely understood. It has been reported PIK3C2A is required for clathrin assembl [25], clathrin-mediated trafficking [25], ATP-dependent priming of neurosecretory granule exocytosis [26], contraction of vascular smooth muscle cells [27], and proliferation and migration of endothelial cells [7, 8]. But little is known about its role in tumor progression. In this study, we found PIK3C2A was frequently down-regulated in HCC cells and tissues, and confirmed the clinical significance of PIK3C2A as an independent prognostic marker for HCC patients after liver resection. Moreover, low PIK3C2A expression was closely associated with elevated LRP6 phosphorylation and Wnt/β-catenin pathway activation in clinical HCC tissues. Moreover, loss of PIK3C2A promoted cell invasion and migration in vitro and HCC metastasis in vivo, and these knockdown phenotypes could be rescued by exogenous expression of PIK3C2A. Notably, we further confirmed hyperactivation of the LRP6/β-catenin pathway in mouse pulmonary metastatic lesions after PIK3C2A knockdown, supporting that this signaling axis drives HCC metastatic progression in vivo. To our best knowledge, it is the first study that intensively evaluate the inhibitory role of PIK3C2A in HCC metastasis. And we are also the first to find PIK3C2A suppresses Wnt/β-catenin signaling via inhibiting phosphorylation of LRP6. Additionally, validation in HepG2 cells, which harbor constitutively active β-catenin, further demonstrated that PIK3C2A negatively regulates LRP6 phosphorylation, β-catenin nuclear translocation, and Wnt target gene expression in a dose‑dependent manner, independent of downstream CTNNB1 mutation status. And yet there still exist some potential problems to be further discussed and explored.

As we all know, in the 16th century, Copernicus propounded heliocentric theory, which differed from the geocentric theory and caused a profound influence on human knowledge. Though such a difference, we may not call this contradiction. We will take it as the development of science. This story coincides with our discovery. Stanley K.L. Ng et al. [28] reported ablation of PIK3C2A suppresses hepatoma cell proliferation. However, we found loss of PIK3C2A caused a slight but significant increase in HCC cell proliferation and tumor growth, and its major role was in HCC metastasis. We gave careful consideration to the difference and found the probable reasons. First of all, the HCC cell line they used differed from ours. In this study we adopted SMMC7721 and PLC/PRF/5, which were widely recognized and used in many HCC researches [29–31], while the Mahlavu they used was seldom adopted. In addition, the study by Winfried Elis et al. [32] also supported our result. They found though in many kinds of tumor cell lines knocking down PIK3C2A showed a significant reduction in cell proliferation and viability, that in HCC cell line Hep G2 was different. Hep G2 was also widely recognized and adopted in many studies [33, 34]. In the heat map, cell viability of Hep G2 with PIK3C2A knocked down compared with control was in green, which meant “unchanged or up-regulated”. Secondly, Stanley’s research only used one HCC cell line Mahlavu, and their results were not validated by in vivo assay. Therefore, it could only be concluded from their study that ablation of PIK3C2A suppresses hepatoma cell line Mahlavu proliferation, not HCC. All in all, more importantly, they did not investigate the role of PIK3C2A in HCC metastasis, and we found the principal role of PIK3C2A in HCC was to facilitate metastasis, while promoting proliferation was in minor position. Furthermore, our discovery might be regarded as the development from theirs, not contradiction, like Copernicus’s heliocentric theory. Notably, one previous study reported that PIK3C2A mRNA can act as a miR-124 sponge to elevate CD151 expression and enhance HCC cell migration and invasion, implying a pro-metastatic role at the RNA level, which also indicated a prominent regulatory effect of PIK3C2A on tumor growth [9]. These observations appear to be inconsistent with our findings that PIK3C2A functions as a metastasis suppressor and exerts only a marginal influence on HCC cell proliferation and tumor growth. Such discrepancies can be well interpreted by the dual molecular pattern of PIK3C2A and the high heterogeneity of HCC. On the one hand, PIK3C2A plays distinct roles at different molecular layers: its mRNA serves as a ceRNA to facilitate HCC malignant progression, whereas its protein kinase activity remodels lipid metabolism and inhibits LRP6-mediated Wnt/β-catenin signaling to restrain HCC metastasis. It is widely acknowledged that a single gene can exert disparate or even opposing functions at the transcriptional and protein functional levels in cancers. On the other hand, accumulating evidence has confirmed that HCC is a highly heterogeneous malignancy with variable genetic backgrounds, pathological subtypes and intrinsic signaling activity, which profoundly shapes the context‑dependent roles of key genes [35]. By contrast, based on multiple well‑recognized HCC cell lines (SMMC7721 and PLC/PRF/5) and two independent clinical cohorts covering SHCC, SLHCC and NHCC subtypes, our in vitro functional assays, in vivo metastatic models and rescue experiments consistently support that the dominant and overall role of PIK3C2A in HCC is suppressing metastatic progression, with only a secondary minor effect on tumor growth. Collectively, the ultimate biological function of PIK3C2A in HCC is coordinately determined by its dual molecular regulation, tumor heterogeneity, cell intrinsic signaling landscape, and pathological subtype features.

Moreover, we found PIK3C2A could inhibit Wnt/β-catenin signaling. Wnt/β-catenin signaling plays a critical role in a variety of biological process, including cell proliferation, migration, polarity establishment and stem cell self-renewal [36]. Deregulation of Wnt/β-catenin signaling is associated with various cancer development. It has been observed this deregulated Wnt/β-catenin signaling occurred in about 95% of HCC [37]. One or more of 19 known extracellular soluble secreted Wnt ligands initiate this signaling by interacting with cell surface receptors Frizzled (Fz) and LRP6. Subsequent recruitment of the downstream signal mediators Dvl to Fz and Axin to LRP6 results in disassembly of the β-catenin destruction complex consisting of Axin, adenomatous polyposis coli (APC), GSK-3β and casein kinase 1 (CK1), ultimately leading to the nuclear translocation of β-catenin. Binding of β-catenin with the transcription factors of the T-cell factor / lymphoid enhancer-binding factor (TCF/LEF) activates the transcription of Wnt target genes, such as c-Myc, Cyclin D1 and MMP7, which then up-regulate cell proliferation, invasion and migration [21]. In this process, production of PI(4,5)P_2_ leads to phosphorylation of LRP6 at Thr1479 and Ser1490, followed by the formation of Wnt-induced LRP6 aggregates known as signalosomes [23, 38]. In the current study we found loss of PIK3C2A increased the production of PI(4,5)P_2_ and thus activated phosphorylation of LRP6. PI and PI(4)P are both preferred substrates of PIK3C2A, therefore generating PI(3)P and PI(3,4)P_2_ [4, 6]. Without the effect of PIK3C2A on PI(4)P, the generation of PI(3,4)P_2_ decreased, and hence the production of PI(4,5)P_2_ by PIP5KI functioning on PI(4)P increased. And that is why Wnt/β-catenin signaling was activated by loss of PIK3C2A. Additionally, we speculated there should be a decrease in PI(3)P and it might also play a role in the activation. This speculation needs to be further explored.

Previously, we have found a specific subtype of HCC, which is large in size and of single lesion, but had similar outcomes with SHCC, and we characterized them as solitary large hepatocellular carcinoma (SLHCC) [18]. Sufficient evidence indicates that different types of HCCs exhibit different molecular and biologic characteristics, which dictates the clinical and pathological phenotype of HCC [39]. In the past few years, we have investigated the molecular and biological characteristics of SLHCC, and found miR-140-5p [40], miR-331-3p [17], miR-188-5p [41], ras homologous gene C (RhoC) [33], etc. were distinctly expressed among SLHCC, SHCC and NHCC. And in this current study, we also found PIK3C2A had different expression patterns in SLHCC, SHCC and NHCC, indicating it might be used to distinguish the clinical subtypes of HCCs. More importantly, we also identified the role PIK3C2A in HCC metastasis from function and mechanism. We found forced re-expression of PIK3C2A in knockdown HCC cells could inhibit their invasion and migration, alluding its anticancer effect and potential for future transformation application in HCC therapy.

In summary, our study demonstrated that PIK3C2A, frequently down-regulated in HCC, associated with aggressive tumor phenotypes, was an independent prognostic indicator for HCC patients after hepatic resection. In addition, the in vitro and in vivo assays validated the inhibitory role of PIK3C2A in HCC metastasis, which brings about a new understanding. Meanwhile, we further verified this inhibitory role was through suppressing Wnt/β-catenin signaling via directly targeting phosphorylation of LRP6. Notably, while our 10-pathway reporter array efficiently identified the dominant Wnt/β-catenin pathway, we acknowledge that this targeted screening strategy has inherent limitations and may not capture all potential signaling networks regulated by PIK3C2A. Future investigations employing unbiased transcriptomic or proteomic profiling will be valuable to achieve a more comprehensive landscape of downstream pathways. Therefore, our study suggests that PIK3C2A can be used as a valuable prognostic biomarker, and strategies designed to up-regulate PIK3C2A may provide a promising method to alleviate HCC progression.

## Supplementary Information

Below is the link to the electronic supplementary material.

Supplementary Material 1.

Supplementary Material 2: Tables S1-S10.

Supplementary Material 3: Figure S1.

Supplementary Material 4 : Figure S2.

Supplementary Material 5: Figure S3.

Supplementary Material 6: Figure S4.

Supplementary Material 7: Figure S5.

## Data Availability

Included in the paper or Supplementary Information (for raw data, not summary data such as means and variances). All data supporting the findings of this study are available within the paper and its Supplementary Information. Microsatellite primer sequences are provided in Supplementary Tables, along with original reference describing the microsatellites used in this study.

## Abbreviations

HCC: Hepatocellular carcinoma
PIK3C2A: Phosphoinositide-3-Kinase, Class 2, Alpha Polypeptide
SLHCC: Solitary large hepatocellular carcinoma
SHCC: Small hepatocellular carcinoma
NHCC: Nodular hepatocellular carcinoma
ANLT: Adjacent non-tumorous liver tissue
mRNA: Messenger RNA
RT-PCR: Reverse-transcription polymerase chain reaction
qRT-PCR: Quantitative Real-time PCR
OS: Overall survival
DFS: Disease-free survival
MMP7: Matrix metalloproteinase 7
GSK-3β: Glycogen synthase kinase 3β
LRP6: Low-density lipoprotein receptor-related protein 6
Dvl: Dishevelled
PI: Phosphatidylinositol
PI(3)P: phosphatidylinositol 3-phosphate
PI(4)P: Phosphatidylinositol 4-phosphate
PI(3,4)P_2_: Phosphatidylinositol (3,4)
