# Supplementary material for: Novel role of the class II α-isoform of phosphatidylinositol 3-kinase in hepatocellular carcinoma: suppressing metastasis by targeting Wnt/β-catenin signaling via inhibiting phosphorylation of LRP6
Source: Cancer Cell Int. 2026 Jun 22;26:297. doi: 10.1186/s12935-026-04368-2 (PMC13520445; doi:10.1186/s12935-026-04368-2)
Supplement: Supplementary file 1 [file 12935_2026_4368_MOESM1_ESM.docx]

**Novel role of the class II α-isoform of phosphatidylinositol 3-kinase in hepatocellular carcinoma: suppressing metastasis by targeting Wnt/β-catenin signaling via inhibiting phosphorylation of LRP6**

**Supplementary data**

**Supplementary Materials and Methods**

**Cell lines and cell culture**

In this study, human HCC cell lines, PLC/PRF5 and HepG2 cells were purchased from the American Type Culture Collection (ATCC, Manassas, VA). HCCLM3 was kindly provided by the Liver Cancer Institute of Fudan University, Shanghai, China, where it was established. SMMC7721, Huh7 and immortalized human normal liver cells L02 were purchased from the Cell Bank of Typical Culture Preservation Committee of Chinese Academy of Science, Shanghai, China. The cells’ characters were described in our previous work (1). These cells were cultured in Dulbecco’s modified Eagle’s medium supplemented with 10% fetal bovine serum and antibiotics at 37°C with 5% CO2.

**RNA extraction and semi-quantitative RT-PCR**

TRIzol^®^ Reagent (Life Technologies, Carlsbad, CA) was used to isolate total RNA from frozen patient samples and cell lines according to the manufacturer’s protocol. cDNA was synthesized using the universal cDNA synthesis kit (Toyobo, tokyo, JP). The RNA was then reverse-transcribed to obtain cDNA by the universal cDNA synthesis kit (Toyobo, tokyo, JP) at 37°C for 50 min. PCR products were separated by electrophoresis on 1.2% agarose gels and were visualized under ultraviolet light after ethidium bromide staining. All quantifications were normalized to the level of endogenous GAPDH as a control. The detailed procedure was as previously described (1). The primer sequences are listed in the Supplementary Table 7.

**Real-Time Quantitative Polymerase Chain Reaction (qRT-PCR)**

The cDNA was subjected to quantitative real-time PCR (qRT-PCR) using the SYBR Green PCR Kit (Roche Life Sciences, Switzerland) and the assay was performed on an PRISM 7300 Sequence Detection System (Applied Biosystems, CA). GAPDH was used as an internal control. The relative levels of expression were quantified and analyzed. The experiments were done in triplicate. The primers were all synthesized and bought from Sangon Biotech (Shanghai, China). The primer sequences are listed in the Supplementary Table 7.

**Western Blot**

Total proteins were extracted with RIPA lysis buffer and separated by SDS-PAGE and then transferred to the PVDF membrane (Roche Life Sciences, Switzerland). The membrane were blocked with 5% skimmed milk and incubated with the appropriate antibody. The antigen-antibody complex on the membrane was detected with enhanced chemiluminescence regents (Thermo Scientific, Waltham, MA). The antibodies are listed in the Supplementary Table 8.

**Immunohistochemistry**

Immunohistochemical staining for tissue was performed using the polymer HRP detection system (Zhongshan Goldenbridge Biotechnology) on formalin-fixed, paraffin-embedded tissue sections that had been cut to 4-µm thickness as described previously (2). The paraffin sections were dewaxed and antigen retrieval with 0.01 M sodium citrate buffer (pH 6.0), followed with 3 % hydrogen peroxide incubated for 20 min at room temperature to block endogenous peroxidase, next with 10 % donkey serum blocking for 30 min. Primary antibodies were incubated overnight at 4 ℃ in a humidified chamber, followed by HRP conjugated secondary antibody incubation for 30 minutes at room temperature. Antibody binding was detected by DAB and reaction was stopped by immersion of tissue sections in distilled water once brown color appeared. Tissue sections were counterstained by hematoxylin, dehydrated in graded ethanols and mounted. Appropriate positive and negative controls were included for each run of IHC. The antibodies were listed in the Supplementary Table 8.The positive level of immunohistochemical staining was scored as described (3). Patients with different positive level of PIK3C2A expression in HCC specimens was divided into a low expression group (negative or weak) and a high expression group (moderate or strong) for further analysis (Fig. S1A). The representative images of different positive level were shown in Fig. 1E. Moreover, to further assess the significance of PIK3C2A loss and gain in tumor compared with corresponding non-tumorous liver background, patients were divided into two groups: PIK3C2A loss group, whose tumor showed lower positive level than ANLT, and PIK3C2A non-loss group, whose tumor showed higher or equivalent positive level compared to ANLT (Fig. S1B).

**Immunofluorescence for cells**

Cells grow at glass coverslips, and then were fixed with 4% paraformaldehyde in phosphate-buffered saline (PBS) with 0.2% Triton. Cells were then blocked for an hour with 1% bovine serum albumin (BSA) followed by incubation with primary antibody overnight at 4°C. Cells were washed and incubated with appropriate secondary antibody and DAPI. The slides were imaged using an inverted fluorescence microscope DMI4000B (Leica). The antibodies are listed in the Supplementary Table 8 and 9.

**Transfection**

PIK3C2A knockdown lentivirus and its negative control lentivirus were purchased from GenePharma (Suzhou, China). Lentivirus containing short hairpin RNAs (shRNA) targeting PIK3C2A was transfected into SMMC7721 and PLC/PRF5 cells according to the manufacturer’s instructions. And one of the sequences (AAGGTTGGCACTTACAAGAAT) was referred as Kazuaki Yoshioka et al(4) used in their study, which was of the highest silencing efficiency and specificity. Cells transfected with negative control sequence were used as controls. Puromycin (final concentration: 2μg/ml) was used to select stable clones.

The adenovirus containing mutated ORF nucleotide sequence of PIK3C2A and its negative control for rescue experiment were purchased from Vigenebio (Jinan, China) and transfected into treated cells according to the manufacturer’s instructions. And the sustaining time of rescued PIK3C2A expression was also measured by Western Blot.

LRP6 knockdown lentivirus was purchased from GenePharma (Suzhou, China) and transfected into HCC cells according to the manufacturer’s instructions. The silencing efficiency of each shRNA was assessed by qRT-PCR.

The above used sequences are listed in the Supplementary Table 10.

***In vitro* cell behavior assays**

Cell wound-healing assay was performed in 6-well plates. When cells grew approximately to 100% confluence in culture medium DMEM with 10% FBS, they were preincubated with Mitomycin-C (Sigma, St. Louis, MO, 10 μg/ml) for 1h at 37 °C (5) to suppress cell proliferation, next rinsed with phosphate-buffered saline (PBS), and then starved for 24 hours in serum-free medium. A sterile 10 μL pipette tip was used to create three separate, parallel wounds. The speed of wound closure was imaged after 48 hours with an inverted microscope TE-2000S (Nikon) and assessed the rate of closure.

For the Transwell migration assay, 5×10^4^ cells in serum-free medium were seeded into the upper chamber of 8-μm Transwell inserts (BD Biosciences, Franklin Lakes, NJ). And medium containing 10% bovine serum albumin was in the lower chamber. After several hours of incubation at 37°C, cells in the upper chamber were removed carefully and cells adhering to the underside of the membrane were stained with 0.1% crystal violet (Beyotime Institute of Biotechnology) and 20% methanol. The number of cells was counted under an inverted microscope (Nikon).

For the Transwell invasion assay, 1×10^5^ cells in serum-free medium were seeded into the upper chamber of 8-μm Transwell inserts with a matrigel coated membrane (BD Biosciences, Franklin Lakes, NJ), while medium containing 10% bovine serum albumin was in the lower chamber. After several hours of incubation at 37°C, gel and cells in the upper chamber were removed carefully and cells adhering to the underside of the membrane were stained with 0.1% crystal violet (Beyotime Institute of Biotechnology) and 20% methanol. The number of cells was counted under an inverted microscope (Nikon).

For cell proliferation assay, 5000 cells were seeded into each well of 96-well plates. Six wells of each group were counted by using hemacytometer (BIO-RAD, CA, USA) (6). For colony formation assay, cells were seeded into 6-well plates at a density of 500 cells / well and cultured for 2 weeks at 37°C. The numbers of colonies per dish were counted after staining with crystal violet (Beyotime Institute of Biotechnology). Only positive colonies (diameter > 40 um) in the dishes were counted and compared (7).

F-actin fluorescent staining was performed for cytoskeleton analysis. Cells grow at glass coverslips, and then were washed with preheated PBS and fixed for 5 minutes in 3.7% formaldehyde solution in PBS. Then washed extensively in PBS and dehydrated with acetone, permeabilized with 0.1% Triton in PBS, and washed again in PBS. Cells were stained with a 50 mg/ml Phalloidin–Tetramethylrhodamine B isothiocyanate (Sigma, St. Louis, MO) solution in PBS for 40 minutes at room temperature. Wash several times with PBS to remove unbound phalloidin conjugate. Finally cells were incubated with appropriate DAPI and washed. The slides were imaged by using an inverted fluorescence microscope DMI4000B (Leica).

All of the above experiments were performed in triplicate.

**Supplementary References:**

1.	Chang RM, Yang H, Fang F, Xu JF, Yang LY. MicroRNA-331-3p promotes proliferation and metastasis of hepatocellular carcinoma by targeting PH domain and leucine-rich repeat protein phosphatase. Hepatology 2014;60:1251-63.

2.	Wu F, Yang L, Li Y, Ou D, Chen D, Fan C. Novel role for epidermal growth factor-like domain 7 in metastasis of human hepatocellular carcinoma. Hepatology 2009;50:1839-50.

3.	Shimizu M, Saitoh Y, Itoh H. Immunohistochemical staining of Ha-ras oncogene product in normal, benign, and malignant human pancreatic tissues. Hum Pathol 1990;21:607-12.

4.	Yoshioka K, Yoshida K, Cui H, Wakayama T, Takuwa N, Okamoto Y, et al. Endothelial PI3K-C2α, a class II PI3K, has an essential role in angiogenesis and vascular barrier function. Nat Med 2012;18:1560-69.

5.	Pullar CE, Chen J, Isseroff RR. PP2A activation by beta2-adrenergic receptor agonists: novel regulatory mechanism of keratinocyte migration. J Biol Chem 2003;278:22555-62.

6.	Fang F, Chang RM, Yu L, Lei X, Xiao S, Yang H, et al. MicroRNA-188-5p suppresses tumor cell proliferation and metastasis by directly targeting FGF5 in hepatocellular carcinoma. J Hepatol 2015;63:874-85.

7.	Garcia-Echeverria C, Pearson MA, Marti A, Meyer T, Mestan J, Zimmermann J, et al. In vivo antitumor activity of NVP-AEW541-A novel, potent, and selective inhibitor of the IGF-IR kinase. Cancer Cell 2004;5:231-39.

**Figure legends**

**Figure 1. PIK3C2A is frequently down-regulated in human HCC cells and tissues.** (A) PIK3C2A is down-regulated in human HCC cells and 30 paired tissues analyzed by qRT-PCR. (B) PIK3C2A is down-regulated in human HCC cells analyzed by RT-PCR and Western Blot. (C) PIK3C2A is down-regulated in human HCC tissues analyzed by RT-PCR and Western Blot. (D) Representative immunohistochemistry (IHC) images of PIK3C2A expression in HCC and ANLT from NHCC, SLHCC and SHCC. Magnifications: ×100, ×400. Bar graph shows statistics for staining intensity of NHCC, SLHCC and SHCC in training and validation cohorts. (E) Representative IHC images of different staining intensity of PIK3C2A in HCC and representative IHC images of PIK3C2A expression in ANLT. Magnifications: ×100, ×400. (F) Representative IHC staining of LRP6, PIK3C2A, and β-catenin in paired HCC and ANLT samples.(G) Quantification of IHC staining intensity for LRP6, PIK3C2A, and β-catenin. (H) Western blot analysis of LRP6, p-LRP6, p-GSK3β, total β-catenin, and nuclear β-catenin in paired HCC and ANLT tissues. (I) Quantitative analysis of Western blot results for LRP6, p-LRP6, p-GSK3β, total β-catenin, and nuclear β-catenin. **P* < 0.05, ***P* < 0.01, ****P* < 0.001 vs. ANLT.

**Figure 2. Low expression of PIK3C2A in HCC tissues is associated with aggressive clinicopathological features and shorter postoperative survival rate.** (A) Representatives IHC images of PIK3C2A loss / non-loss (retain/gain) cases. Magnifications: ×100, ×400. (B) The overall survival (OS) and disease-free survival (DFS) of HCC patients with low / high PIK3C2A expression and PIK3C2A loss / non-loss in training cohort and validation cohort. Survival curve was calculated with the log-rank test and *P* value was shown in each panel respectively.

**Figure 3. Loss of PIK3C2A promotes invasion and migration of HCC cells *in vitro* and tumor metastasis *in vivo*.** (A) Effects of PIK3C2A silencing on cell invasion and migration using Transwell invasion / migration assay. Representative images of invasive or migrated cells are shown (left panel). Magnification: ×100. Results represented the mean ± SD in triplicate using bar graph (right panel). ***P*<0.01. (B) Effects of PIK3C2A silencing on cytoskeleton analyzed by F-actin fluorescent staining. Original magnification: ×400. (C) Effects of PIK3C2A silencing on in vivo tumor growth and metastasis. (C1) Representative bioluminescent imaging of the different groups is shown at 6 weeks after orthotopic implantation (left panel). Blue arrow indicates lung metastatic nodule and red arrow indicates intrahepatic metastasis. The growth curve showed the total flux of orthotopic tumors in each group every week detected by IVIS indicating the growth rate of tumors (right panel). **P*<0.05, ***P*<0.01. (C2) Representative pictures of intrahepatic metastasis (upper panel). Yellow arrow indicates intrahepatic metastasis. Bar graph shows statistics for the percentage of mice with or without metastatic nodules in the livers (lower panel). (C3) Representative pictures of pulmonary metastasis using Hematoxylin and eosin (H&E) staining (upper panel). Magnifications: ×100, ×400. Bar graph shows statistics for the percentage of mice with or without metastatic nodules in the lungs (lower panel). (D) Representative Western blot images showing the expression of PIK3C2A, LRP6, p-LRP6, total and nuclear β-catenin, Cyclin D1, c-Myc, and MMP7 in shCon and shPIK3C2A groups. β-actin was used as the loading control. (E) Quantitative analysis of the Western blot results. Data are presented as mean ± SD; **P* < 0.05, ***P* < 0.01, ****P* < 0.001 vs. shCon.

**Figure 4. PIK3C2A knockdown phenotype in HCC cells can be rescued by exogenous expression of PIK3C2A.** (A) Effects of PIK3C2A silencing and rescue on cell invasion and migration using Transwell invasion / migration assay. Representative images of invasive or migrated cells are shown (left panel). Magnification: ×100. Results represented the mean ± SD in triplicate using bar graph (right panel). ***P*<0.01. (B) Effects of PIK3C2A silencing and rescue on cytoskeleton analyzed by F-actin fluorescent staining. Original magnification: ×400.

**Figure 5. Loss of PIK3C2A activates Wnt/β-catenin signaling and abrogates its direct inhibition on LRP6 phosphorylation.** (A) 10-Pathway Reporter Array showed the signaling change in PIK3C2A-interfered cells. (B) Protein levels of c-Myc, Cyclin D1 and MMP7 detected by Western Blot were shown in indicated cells. (C) Protein levels of phosphorylated-β-catenin, active β-catenin and total β-catenin detected by Western Blot were shown in indicated cells. (D) Protein levels of cytoplasmic and nuclear β-catenin detected by Western Blot were shown in indicated cells. (E) Protein levels of normal and phosphorylated form of LRP6 and GSK-3β detected by Western Blot were shown in indicated cells. (F) Immunofluorescence staining of β-catenin in indicated cells. Original magnification: ×100. (G) Representative results of protein lipid overlay assay of the PI(3,4)P_2_ and PI(4,5)P_2_ (left panel). Bar graph shows the relative level of PI(3,4)P_2_ and PI(4,5)P_2_ in shPIK3C2A cells compared with shcon cells (right panel). (H) Representative Western blot images of p-LRP6, LRP6, total β-catenin, nuclear β-catenin, Cyclin D1, c-Myc, and Axin2 in Control, shPIK3C2A, and AdPIK3C2A groups. β-actin was used as the loading control. (I) Quantitative analysis of the Western blot results. Data are presented as mean ± SD; **P* < 0.05, ***P* < 0.01, ****P* < 0.001.

**Figure 6. Inhibiting phosphorylation of LRP6 by LRP6 knockdown mimics impact of PIK3C2A on cell invasion and migration.** (A) Protein levels of LRP6, p-LRP6, GSK-3β, p-GSK-3β and MMP7 detected by Western Blot were shown in indicated cells. shLRP6^1^ and shLRP6^2^ were the sequence of shLRP6-3 and shLRP6-1, which caused significant decrease of about 50% and 85% respectively in the expression of LRP6 mRNA validated by qRT-PCR. (B) Transwell invasion / migration assay were conducted in indicated cells to explore effects of inhibiting phosphorylation of LRP6 in different levels if can mimic the role of PIK3C2A on cell invasion and migration using. Representative images of invasive or migrated cells are shown (left panel). Magnification: ×100. Results represented the mean ± SD in triplicate using bar graph (right panel). **P*<0.05, ***P*<0.01. shLRP6^1^ and shLRP6^2^ were the sequence of shLRP6-3 and shLRP6-1, which caused significant decrease of about 50% and 85% respectively in the expression of LRP6 mRNA validated by qRT-PCR.

**Supplementary Figure Legends**

**Figure S1. Flow diagram of patients included in study.** (A) Patients were dichotomized into PIK3C2A high expression group and low expression group. (B) Patients were dichotomized into PIK3C2A loss group (T < N) and non-loss group (T ≥ N).

**Figure S2. Loss of PIK3C2A promotes migration and proliferation of HCC cells *in vitro*.** (A) Silencing efficiency of PIK3C2A in mRNA level by shPIK3C2A-1 and shPIK3C2A-2 in HCC cells were identified by qRT-PCR. And shPIK3C2A-2 were chosen for further research. ***P*<0.01. (B) Silencing efficiency of PIK3C2A in protein level by shPIK3C2A-2 in HCC cells were further validated by Western Blot. (C) Effects of PIK3C2A silencing on cell migration using wound-healing assay. Representative images of invasive or migrated cells are shown (left panel). Magnification: ×100. Results represented the mean ± SD in triplicate using bar graph (right panel). ***P*<0.01. (D) Effects of PIK3C2A silencing on cell proliferation using colony formation assay. Representative images of colony formation are shown (left panel). Results represented the mean ± SD in triplicate using bar graph (right panel). **P*<0.05, ***P*<0.01. (E) Effects of PIK3C2A silencing on cell proliferation analyzed by cell proliferation assay with using hemacytometer. **P*<0.05, ***P*<0.01.

**Figure S3. Loss of PIK3C2A promotes HCC growth and metastasis *in vivo*.** (A) The expression of PIK3C2A in mouse tumors of different groups was verified by Western Blot. (B) Effects of PIK3C2A silencing on *in vivo* tumor growth. Liver orthotopic tumors from each indicated groups are shown (left panel). Tumor volumes of tumors were shown by bar graph (right panel). **P*<0.05. (C) Representative images of IVIS showed the metastatic nodule in harvested internal organs (yellow arrows indicate tumor nodules).

**Figure S4. The increased proliferation by PIK3C2A knockdown in HCC cells can be inhibited by exogenous expression of PIK3C2A.** (A) The expression of PIK3C2A in shPIK3C2A cells of different time after adenovirus transfection containing mutated ORF nucleotide sequence of PIK3C2A analyzed by Western Blot. (B) The expression of PIK3C2A in indicated cells detected by Western Blot to further validate its expression rescued. Proteins of shPIK3C2A+Advec group and shPIK3C2A+AdPIK3C2A group were collected at 48h after adenovirus transfection. (C) Effects of PIK3C2A silencing and rescue on cell proliferation using colony formation assay. Representative images of colony formation are shown (left panel). Results represented the mean ± SD in triplicate using bar graph (right panel). **P*<0.05, ***P*<0.01. (D) Effects of PIK3C2A silencing and rescue on cell proliferation analyzed by cell proliferation assay with using hemacytometer. **P*<0.05, ***P*<0.01.

**Figure S5. Relative expression of LRP6 in transfected cells compared with untreated cells was measured by qRT-PCR.** Results represented the mean ± SD in triplicate using bar graph (left panel). The detailed results were shown in right panel. The red column indicated the two chosen anti-sequences to perform functional assays. ***P*<0.01.
