# Supplementary material for: Novel role of the class II α-isoform of phosphatidylinositol 3-kinase in hepatocellular carcinoma: suppressing metastasis by targeting Wnt/β-catenin signaling via inhibiting phosphorylation of LRP6
Source: Cancer Cell Int. 2026 Jun 22;26:297. doi: 10.1186/s12935-026-04368-2 (PMC13520445; doi:10.1186/s12935-026-04368-2)
Supplement: Supplementary file 2 [file 12935_2026_4368_MOESM2_ESM.docx]

**Supplementary tables**

**Supplementary table 1. Clinicopathologcal Characteristics of Patients in Training Cohort and in Validation Cohort**

| Clinicopathologic Variable | Counts | | *P* |
| --- | --- | --- | --- |
|  | Training cohort | Validation cohort |  |
| Gender |  |  |  |
| Female | 21(16.2%) | 15(21.1%) |  |
| Male | 109(83.8%) | 56(78.9%) | 0.379 |
| Age(year) |  |  |  |
| ≤60 | 87(66.9%) | 49(69.0%) |  |
| ＞60 | 43(33.1%) | 22(31.0%) | 0.762 |
| AFP |  |  |  |
| <20ng/ml | 61(46.9%) | 38(53.5%) |  |
| ≥20ng/ml | 69(53.1%) | 33(46.5%) | 0.371 |
| HBsAg |  |  |  |
| Negative | 21(16.2%) | 10(14.1%) |  |
| Positive | 109(83.8%) | 61(85.9%) | 0.698 |
| Liver cirrhosis |  |  |  |
| Absence | 49(37.7%) | 22(31.0%) |  |
| presence | 81(62.3%) | 49(69.0%) | 0.342 |
| Tumor size(cm) |  |  |  |
| ≤5 | 57(43.8%) | 33(46.5%) |  |
| ＞5 | 73(56.2%) | 38(53.5%) | 0.720 |
| Tumor nodule number |  |  |  |
| Solitary | 79(60.8%) | 42(59.2%) |  |
| Multiple(≥2) | 51(39.2%) | 29(40.8%) | 0.823 |
| Capsular formation |  |  |  |
| Presence | 64(49.2%) | 30(42.3%) |  |
| Absence | 66(50.8%) | 41(57.7%) | 0.343 |
| Edmondson-Steiner grade |  |  |  |
| Ⅰ-Ⅱ | 77(59.2%) | 41(57.7%) |  |
| Ⅲ-Ⅳ | 53(40.8%) | 30(42.3%) | 0.838 |
| Venous invasion |  |  |  |
| Absence | 74(56.9%) | 39(54.9%) |  |
| Presence | 56(43.1%) | 32(45.1%) | 0.785 |
| Subtype of HCC |  |  |  |
| SHCC | 31(23.8%) | 18(25.4%) |  |
| SLHCC | 48(36.9%) | 24(33.8%) |  |
| NHCC | 51(39.2%) | 29(40.8%) | 0.906 |
| TNM stage |  |  |  |
| Ⅰ | 55(42.3%) | 26(36.6%) |  |
| Ⅱ | 31(23.8%) | 19(26.8%) |  |
| Ⅲ | 44(33.8%) | 26(36.6%) | 0.731 |
| BCLC stage |  |  |  |
| 0-A | 37(28.5%) | 22(31.0%) |  |
| B | 66(50.8%) | 35(49.3%) |  |
| C | 27(20.8%) | 14(19.7%) | 0.930 |
| Liver function |  |  |  |
| Child-Pugh A | 91(70.0%) | 46(64.8%) |  |
| Child-Pugh B | 39(30.0%) | 25(35.2%) | 0.448 |

**Supplementary table 2. Univariate and Multivariate Analysis of Factors Associated with Overall Survival in Training Cohort**

|  |  | Univariate Analysis | | Multivariate Analysis | |
| --- | --- | --- | --- | --- | --- |
| Variable | n | RR(95%CI) | *P* | RR(95%CI) | *P* |
| Gender |  |  |  |  |  |
| Female | 21 | 1 |  |  |  |
| Male | 109 | 1.403(0.757-2.598) | 0.282 | n.a. | n.a. |
| Age(year) |  |  |  |  |  |
| ≤60 | 87 | 1 |  |  |  |
| ＞60 | 43 | 1.194(0.745-1.913) | 0.461 | n.a. | n.a. |
| AFP |  |  |  |  |  |
| <20ng/ml | 61 | 1 |  |  |  |
| ≥20ng/ml | 69 | 1.526(0.973-2.393) | 0.066 | n.a. | n.a. |
| HBsAg |  |  |  |  |  |
| Negative | 21 | 1 |  |  |  |
| Positive | 109 | 0.829(0.472-1.456) | 0.514 | n.a. | n.a. |
| Liver cirrhosis |  |  |  |  |  |
| Absence | 49 | 1 |  |  |  |
| Presence | 81 | 1.157 (0.728-1.839) | 0.537 | n.a. | n.a. |
| Tumor size(cm) |  |  |  |  |  |
| ≤5 | 57 | 1 |  |  |  |
| ＞5 | 73 | 0.850(0.545-1.327) | 0.475 | n.a. | n.a. |
| Tumor nodule number |  |  |  |  |  |
| Solitary | 79 | 1 |  | 1 |  |
| Multiple(≥2) | 51 | 3.098( 1.964-4.887) | **<0.001** | 2.420 (1.476-3.965) | **<0.001** |
| Capsular formation |  |  |  |  |  |
| Presence | 64 | 1 |  | 1 |  |
| Absence | 66 | 1.771(1.126-2.785) | **0.013** | 0.628(0.313-1.262) | 0.192 |
| Edmondson-Steiner grade |  |  |  |  |  |
| Ⅰ-Ⅱ | 77 | 1 |  |  |  |
| Ⅲ-Ⅳ | 53 | 1.411(0.904-2.203) | 0.130 | n.a. | n.a. |
| Venous invasion |  |  |  |  |  |
| Absence | 74 | 1 |  | 1 |  |
| Presence | 56 | 3.290（2.078-5.211) | **<0.001** | 2.982(1.286-6.914) | **0.011** |
| TNM stage |  |  |  |  |  |
| Ⅰ | 55 | 1 |  | 1 |  |
| Ⅱ | 31 |  |  |  |  |
| Ⅲ | 44 | 1.849(1.439-2.375) | **<0.001** | 1.039(0.709-1.524) | 0.843 |
| BCLC stage |  |  |  |  |  |
| 0-A | 37 | 1 |  |  |  |
| B | 66 |  |  |  |  |
| C | 27 | 1.138(0.818-1.582) | 0.444 | n.a. | n.a. |
| Liver function |  |  |  |  |  |
| Child-Pugh A | 91 | 1 |  |  |  |
| Child-Pugh B | 39 | 0.721(0.434-1.199) | 0.207 | n.a. | n.a. |
| PIK3C2A expression |  |  |  |  |  |
| Higher | 67 | 1 |  | 1 |  |
| Lower | 63 | 2.448(1.553-3.858) | **<0.001** | 1.798(1.098-2.945) | **0.020** |

**Supplementary table 3. Univariate and Multivariate Analysis of Factors Associated with Disease-Free Survival in Training Cohort**

|  |  | Univariate Analysis | | Multivariate Analysis | |
| --- | --- | --- | --- | --- | --- |
| Variable | n | RR(95%CI) | *P* | RR(95%CI) | *P* |
| Gender |  |  |  |  |  |
| Female | 21 | 1 |  |  |  |
| Male | 109 | 1.535(0.871-2.704) | 0.138 | n.a. | n.a. |
| Age(year) |  |  |  |  |  |
| ≤60 | 87 | 1 |  |  |  |
| ＞60 | 43 | 1.187(0.781-1.804) | 0.423 | n.a. | n.a. |
| AFP |  |  |  |  |  |
| <20ng/ml | 61 | 1 |  |  |  |
| ≥20ng/ml | 69 | 1.359(0.914-2.022) | 0.130 | n.a. | n.a. |
| HBsAg |  |  |  |  |  |
| Negative | 21 | 1 |  |  |  |
| Positive | 109 | 0.983(0.575-1.680) | 0.950 | n.a. | n.a. |
| Liver cirrhosis |  |  |  |  |  |
| Absence | 49 | 1 |  |  |  |
| Presence | 81 | 1.087(0.713-1.635) | 0.688 | n.a. | n.a. |
| Tumor size(cm) |  |  |  |  |  |
| ≤5 | 57 | 1 |  |  |  |
| ＞5 | 73 | 0.946(0.635-1.407) | 0.783 | n.a. | n.a. |
| Tumor nodule number |  |  |  |  |  |
| Solitary | 79 | 1 |  | 1 |  |
| Multiple(≥2) | 51 | 3.071(2.040-4.625) | **<0.001** | 2.469(1.576-3.867) | **<0.001** |
| Capsular formation |  |  |  |  |  |
| Presence | 64 | 1 |  | 1 |  |
| Absence | 66 | 1.566(1.052-2.332) | **0.027** | 0.646(0.352-1.183) | 0.157 |
| Edmondson-Steiner grade |  |  |  |  |  |
| Ⅰ-Ⅱ | 77 | 1 |  |  |  |
| Ⅲ-Ⅳ | 53 | 1.313(0.881-1.957) | 0.181 | n.a. | n.a. |
| Venous invasion |  |  |  |  |  |
| Absence | 74 | 1 |  | 1 |  |
| Presence | 56 | 2.868(1.910-4.306) | **<0.001** | 2.599(1.240-5.445) | **0.011** |
| TNM stage |  |  |  |  |  |
| Ⅰ | 55 | 1 |  | 1 |  |
| Ⅱ | 31 |  |  |  |  |
| Ⅲ | 44 | 1.767(1.418-2.203) | **<0.001** | 1.035(0.736-1.456) | 0.843 |
| BCLC stage |  |  |  |  |  |
| 0-A | 37 | 1 |  |  |  |
| B | 66 |  |  |  |  |
| C | 27 | 1.287(0.960-1.725) | 0.091 | n.a. | n.a. |
| Liver function |  |  |  |  |  |
| Child-Pugh A | 91 | 1 |  |  |  |
| Child-Pugh B | 39 | 0.848(0.550-1.309) | 0.457 | n.a. | n.a. |
| PIK3C2A expression |  |  |  |  |  |
| Higher | 67 | 1 |  | 1 |  |
| Lower | 63 | 2.396(1.602-3.583) | **<0.001** | 1.908(1.243-2.929) | **0.003** |

**Supplementary table 4. The Correlations of PIK3C2A with Clinicopathological Features of HCC in Validation Cohort**

|  |  | PIK3C2A | |  |
| --- | --- | --- | --- | --- |
| Clinicopathologic Variable | n | Low expression | High expression | *P* |
| Gender |  |  |  |  |
| Female | 15 | 7 | 8 |  |
| Male | 56 | 27 | 29 | 0.915 |
| Age(year) |  |  |  |  |
| ≤60 | 49 | 24 | 25 |  |
| ＞60 | 22 | 10 | 12 | 0.783 |
| AFP |  |  |  |  |
| <20ng/ml | 38 | 15 | 23 |  |
| ≥20ng/ml | 33 | 19 | 14 | 0.128 |
| HBsAg |  |  |  |  |
| Negative | 10 | 6 | 4 |  |
| Positive | 61 | 28 | 33 | 0.408 |
| Liver cirrhosis |  |  |  |  |
| Absence | 22 | 10 | 12 |  |
| presence | 49 | 24 | 25 | 0.783 |
| Tumor size(cm) |  |  |  |  |
| ≤5 | 33 | 14 | 19 |  |
| ＞5 | 38 | 20 | 18 | 0.390 |
| Tumor nodule number |  |  |  |  |
| Solitary | 42 | 15 | 27 |  |
| Multiple(≥2) | 29 | 19 | 10 | **0.013** |
| Capsular formation |  |  |  |  |
| Presence | 30 | 9 | 21 |  |
| Absence | 41 | 25 | 16 | **0.010** |
| Edmondson-Steiner grade |  |  |  |  |
| Ⅰ-Ⅱ | 41 | 18 | 23 |  |
| Ⅲ-Ⅳ | 30 | 16 | 14 | 0.432 |
| Venous invasion |  |  |  |  |
| Absence | 39 | 12 | 27 |  |
| Presence | 32 | 22 | 10 | **0.001** |
| Subtype of HCC |  |  |  |  |
| SHCC | 18 | 7 | 11 |  |
| SLHCC | 24 | 8 | 16 |  |
| NHCC | 29 | 19 | 10 | **0.044** |
| TNM stage |  |  |  |  |
| Ⅰ | 26 | 4 | 22 |  |
| Ⅱ | 19 | 10 | 9 |  |
| Ⅲ | 26 | 20 | 6 | **<0.001** |
| BCLC stage |  |  |  |  |
| 0-A | 22 | 11 | 11 |  |
| B | 35 | 14 | 21 |  |
| C | 14 | 9 | 5 | 0.298 |
| Liver function |  |  |  |  |
| Child-Pugh A | 46 | 19 | 27 |  |
| Child-Pugh B | 25 | 15 | 10 | 0.132 |

**Supplementary table 5. Univariate and Multivariate Analysis of Factors Associated with Overall Survival in Validation Cohort**

|  |  | Univariate Analysis | | Multivariate Analysis | |
| --- | --- | --- | --- | --- | --- |
| Variable | n | RR(95%CI) | *P* | RR(95%CI) | *P* |
| Gender |  |  |  |  |  |
| Female | 15 | 1 |  |  |  |
| Male | 56 | 1.121(0.554-2.269) | 0.751 | n.a. | n.a. |
| Age(year) |  |  |  |  |  |
| ≤60 | 49 | 1 |  |  |  |
| ＞60 | 22 | 1.109(0.581-2.118) | 0.754 | n.a. | n.a. |
| AFP |  |  |  |  |  |
| <20ng/ml | 38 | 1 |  |  |  |
| ≥20ng/ml | 33 | 1.569(0.871-2.826) | 0.134 | n.a. | n.a. |
| HBsAg |  |  |  |  |  |
| Negative | 10 | 1 |  |  |  |
| Positive | 61 | 1.383(0.545-3.513) | 0.495 | n.a. | n.a. |
| Liver cirrhosis |  |  |  |  |  |
| Absence | 22 | 1 |  |  |  |
| Presence | 49 | 0.924(0.491-1.738) | 0.806 | n.a. | n.a. |
| Tumor size(cm) |  |  |  |  |  |
| ≤5 | 33 | 1 |  |  |  |
| ＞5 | 38 | 0.952(0.530-1.711) | 0.870 | n.a. | n.a. |
| Tumor nodule number |  |  |  |  |  |
| Solitary | 42 | 1 |  | 1 |  |
| Multiple(≥2) | 29 | 2.652(1.451-4.847) | **0.002** | 2.834(1.418-5.663) | **0.003** |
| Capsular formation |  |  |  |  |  |
| Presence | 30 | 1 |  | 1 |  |
| Absence | 41 | 2.283(1.226-4.250) | **0.009** | 0.657(0.256-1.681) | 0.381 |
| Edmondson-Steiner grade |  |  |  |  |  |
| Ⅰ-Ⅱ | 41 | 1 |  |  |  |
| Ⅲ-Ⅳ | 30 | 1.307(0.725-2.355) | 0.373 | n.a. | n.a. |
| Venous invasion |  |  |  |  |  |
| Absence | 39 | 1 |  | 1 |  |
| Presence | 32 | 3.503(1.891-6.488) | **<0.001** | 4.509(1.547-13.145) | **0.006** |
| TNM stage |  |  |  |  |  |
| Ⅰ | 26 | 1 |  | 1 |  |
| Ⅱ | 19 |  |  |  |  |
| Ⅲ | 26 | 1.776(1.267-2.490) | **0.001** | 0.837(0.494-1.419) | 0.510 |
| BCLC stage |  |  |  |  |  |
| 0-A | 22 | 1 |  |  |  |
| B | 35 |  |  |  |  |
| C | 14 | 1.089(0.721-1.645) | 0.686 | n.a. | n.a. |
| Liver function |  |  |  |  |  |
| Child-Pugh A | 46 | 1 |  |  |  |
| Child-Pugh B | 25 | 1.345(0.729-2.479) | 0.343 | n.a. | n.a. |
| PIK3C2A expression |  |  |  |  |  |
| Higher | 37 | 1 |  | 1 |  |
| Lower | 34 | 2.855(1.558-5.232) | **0.001** | 1.973(1.021-3.812) | **0.043** |

**Supplementary table 6. Univariate and Multivariate Analysis of Factors Associated with Disease-Free Survival in Validation Cohort**

|  |  | Univariate Analysis | | Multivariate Analysis | |
| --- | --- | --- | --- | --- | --- |
| Variable | n | RR(95%CI) | *P* | RR(95%CI) | *P* |
| Gender |  |  |  |  |  |
| Female | 15 | 1 |  |  |  |
| Male | 56 | 1.530(0.772-3.033) | 0.223 | n.a. | n.a. |
| Age(year) |  |  |  |  |  |
| ≤60 | 49 | 1 |  |  |  |
| ＞60 | 22 | 1.403(0.806-2.440) | 0.231 | n.a. | n.a. |
| AFP |  |  |  |  |  |
| <20ng/ml | 38 | 1 |  |  |  |
| ≥20ng/ml | 33 | 1.262(0.747-2.132) | 0.134 | n.a. | n.a. |
| HBsAg |  |  |  |  |  |
| Negative | 10 | 1 |  |  |  |
| Positive | 61 | 1.010(0.476-2.144) | 0.979 | n.a. | n.a. |
| Liver cirrhosis |  |  |  |  |  |
| Absence | 22 | 1 |  |  |  |
| Presence | 49 | 1.041(0.581-1.864) | 0.893 | n.a. | n.a. |
| Tumor size(cm) |  |  |  |  |  |
| ≤5 | 33 | 1 |  |  |  |
| ＞5 | 38 | 0.990(0.588-1.668) | 0.970 | n.a. | n.a. |
| Tumor nodule number |  |  |  |  |  |
| Solitary | 42 | 1 |  | 1 |  |
| Multiple(≥2) | 29 | 2.763( 1.612-4.734) | **<0.001** | 2.912(1.529-5.547) | **0.001** |
| Capsular formation |  |  |  |  |  |
| Presence | 30 | 1 |  | 1 |  |
| Absence | 41 | 2.539(1.457-4.426) | **0.001** | 1.100(0.503-2.407) | 0.812 |
| Edmondson-Steiner grade |  |  |  |  |  |
| Ⅰ-Ⅱ | 41 | 1 |  |  |  |
| Ⅲ-Ⅳ | 30 | 1.248(0.736-2.116) | 0.411 | n.a. | n.a. |
| Venous invasion |  |  |  |  |  |
| Absence | 39 | 1 |  | 1 |  |
| Presence | 32 | 2.879（1.677-4.941) | **<0.001** | 2.716(1.104-6.681) | **0.030** |
| TNM stage |  |  |  |  |  |
| Ⅰ | 26 | 1 |  | 1 |  |
| Ⅱ | 19 |  |  |  |  |
| Ⅲ | 26 | 1.723(1.283-2.314) | **<0.001** | 0.889(0.560-1.412) | 0.618 |
| BCLC stage |  |  |  |  |  |
| 0-A | 22 | 1 |  |  |  |
| B | 35 |  |  |  |  |
| C | 14 | 1.160(0.804-1.676) | 0.427 | n.a. | n.a. |
| Liver function |  |  |  |  |  |
| Child-Pugh A | 46 | 1 |  |  |  |
| Child-Pugh B | 25 | 1.286(0.746-2.219) | 0.365 | n.a. | n.a. |
| PIK3C2A expression |  |  |  |  |  |
| Higher | 37 | 1 |  | 1 |  |
| Lower | 34 | 2.617(1.536-4.461) | **<0.001** | 1.929(1.089-3.415) | **0.024** |

**Supplementary Table 7. The sequences of PCR primers used in this study**

| Target gene | Application | Sequence |
| --- | --- | --- |
| PIK3C2A | RT-PCR | F: CTGGTTCCTGCTTCCGATAC |
|  |  | R: CAGAGGTCCACAAACAACTGA |
| PIK3C2A | real-time PCR | F: AAACCAACACCGAGCAGTAGAT |
|  |  | R: TCCTCCAAACAAAGAAGTCACA |
| GAPDH | RT-PCR | F: AGAAGGCTGGGGCTCATTTG |
|  |  | R: AGGGGCCATCCACAGTCTTC |
| GAPDH | real-time PCR | F: GTCTCCTCTGACTTCAACAGCG |
|  |  | R: ACCACCCTGTTGCTGTAGCCAA |
| LRP6 | real-time PCR | F: CCCATGCACCTGGTTCTACT |
|  |  | R: CCAAGCCACAGGGATACAGT |

**Supplementary Table 8. List of the antibodies used in this study.**

| Antibody name | Source |
| --- | --- |
| PIK3C2A | Santa Cruz (sc-67306) |
| β-actin | Sigma Aldrich (A5316) |
| c-Myc | Santa Cruz (sc-40) |
| Cyclin D1 | Santa Cruz (sc-753) |
| MMP-7 | Santa Cruz (sc-80205) |
| p-β-catenin | Cell signaling (9561) |
| Active β-catenin | Cell signaling (8814) |
| β-catenin | Santa Cruz (sc-133239) |
| Histone H3.1 | Affinity (AF6358) |
| LRP6 | Cell signaling (2560) |
| p-LRP6 | Cell signaling (2568) |
| GSK-3β | Santa Cruz (sc-377213) |
| p- GSK-3β | Santa Cruz (sc-373800) |
| PI(3,4)P_2_ | Echlon Biosciences (Z-P034b) |
| GST | Sigma (G 1160) |

**Supplementary Table 9. List of the reagents used in this study.**

| Reagent name | Source |
| --- | --- |
| Donkey anti-Mouse IgG (H+L) Cross Adsorbed Secondary Antibody, DyLight 594 conjugate | ThermoFisher Scientific (SA5-10168) |
| Anti-Mouse IgG (whole molecule)–Peroxidase antibody produced in goat | Sigma (A5278) |
| Goat anti-Rabbit IgG-HRP Secondary Antibody | ZSGB-BIO (ZDR-5306) |
| PI(4,5)P_2_ Grip™ | Echlon Biosciences (G-4501) |
| Mitomycin C | Roche (M8170) |
| Phalloidin–Tetramethylrhodamine B isothiocyanate | Sigma (P1951) |
| D-luciferin, potassium salt | Sciencelight (luc001) |
| Polymer HRP Detection System | ZSGB-BIO (PV-9000) |
| Hematoxylin and eosin (H&E) staining kit | Solarbio (G1120) |
| Cignal Finder Cancer 10-Pathway Reporter Array | QIAGEN (CCA-101L) |

**Supplementary Table 10. The sequences of shRNA and cDNA clone used in this study**

| Name | Sequence |
| --- | --- |
| PIK3C2A-shRNA-1 | GGTCTCCATTGACATTGAAGG |
| PIK3C2A-shRNA-2 | AAGGTTGGCACTTACAAGAAT |
| Negative Control | TTCTCCGAACGTGTCACGTTTC |
| LRP6-shRNA-1 | GCAGCCAAATGCCACAAATCC |
| LRP6-shRNA-2 | GGTTCTGACCGTGTAGTATTG |
| LRP6-shRNA-3 | GCAGATATCAGACGAATTTCT |
| LRP6-shRNA-4 | GCACCTGGTTCTACTTCAAGA |
| Mutated (in red) ORF nucleotide sequence of PIK3C2A for rescue experiment | ATGGCTCAGATATCTAGCAACAGCGGATTTAAAGAATGTCCATCTTCACATCCGGAACCAACAAGAGCAAAAGATGTGGACAAAGAAGAAGCATTACAGATGGAAGCAGAGGCTTTAGCAAAACTGCAAAAGGATAGACAAGTGACTGACAATCAGAGAGGCTTTGAGTTGTCAAGCAGCACCAGAAAAAAAGCACAGGTTTATAACAAGCAGGATTATGATCTCATGGTGTTTCCTGAATCAGATTCCCAAAAAAGAGCATTAGATATTGATGTAGAAAAGCTCACCCAAGCTGAACTTGAGAAACTATTGCTGGATGACAGTTTCGAGACTAAAAAAACACCTGTATTACCAGTTACTCCTATTCTGAGCCCTTCCTTTTCAGCACAGCTCTATTTTAGACCTACTATTCAGAGAGGACAGTGGCCACCTGGATTACCTGGGCCTTCCACTTATGCTTTACCTTCTATTTATCCTTCTACTTACAGTAAACAGGCTGCATTCCAAAATGGCTTCAATCCAAGAATGCCCACTTTTCCATCTACAGAACCTATATATTTAAGTCTTCCGGGACAATCTCCATATTTCTCATATCCTTTGACACCTGCCACACCCTTTCATCCACAAGGAAGCTTACCTATCTATCGTCCAGTAGTCAGTACTGACATGGCAAAACTATTTGACAAAATAGCTAGTACATCAGAATTTTTAAAAAATGGGAAAGCAAGGACTGATTTGGAGATAACAGATTCAAAAGTCAGCAATCTACAGGTATCTCCAAAGTCTGAGGATATCAGTAAATTTGACTGGTTAGACTTGGATCCTCTAAGTAAGCCTAAGGTGGATAATGTGGAGGTATTAGACCATGAGGAAGAGAAAAATGTTTCAAGTTTGCTAGCAAAGGATCCTTGGGATGCTGTTCTTCTTGAAGAGAGATCGACAGCAAATTGTCATCTTGAAAGAAAGGTGAATGGAAAATCCCTTTCTGTGGCAACTGTTACAAGAAGCCAGTCTTTAAATATTCGAACAACTCAGCTTGCAAAAGCCCAGGGCCATATATCTCAGAAAGACCCAAATGGGACCAGTAGTTTGCCAACTGGAAGTTCTCTTCTTCAAGAAGTTGAAGTACAGAATGAGGAGATGGCAGCTTTTTGTCGATCCATTACAAAATTGAAGACCAAATTTCCATATACCAATCACCGCACAAACCCAGGCTATTTGTTAAGTCCAGTCACAGCACAAAGAAACATATGCGGAGAAAATGCTAGTGTGAAGGTCTCCATTGACATTGAAGGATTTCAGCTACCAGTTACTTTTACGTGTGATGTGAGTTCTACTGTAGAAATCATTATAATGCAAGCCCTTTGCTGGGTACATGATGACTTGAATCAAGTAGATGTTGGCAGCTATGTTCTAAAAGTTTGTGGTCAAGAGGAAGTGCTGCAGAATAATCATTGCCTTGGAAGTCATGAGCATATTCAAAACTGTCGAAAATGGGACACAGAAATTAGACTACAACTCTTGACCTTCAGTGCAATGTGTCAAAATCTGGCCCGAACAGCAGAAGATGATGAAACACCCGTGGATTTAAACAAACACCTGTATCAAATAGAAAAACCTTGCAAAGAAGCCATGACGAGACACCCTGTTGAAGAACTCTTAGATTCTTATCACAACCAAGTAGAACTGGCTCTTCAAATTGAAAACCAACACCGAGCAGTAGATCAAGTAATTAAAGCTGTAAGAAAAATCTGTAGTGCTTTAGATGGTGTCGAGACTCTTGCCATTACAGAATCAGTAAAGAAGCTAAAGAGAGCAGTTAATCTTCCAAGGAGTAAAACTGCTGATGTGACTTCTTTGTTTGGAGGAGAAGACACTAGCAGGAGTTCAACTAGGGGCTCACTTAATCCTGAAAATCCTGTTCAAGTAAGCATAAACCAATTAACTGCAGCAATTTATGATCTTCTCAGACTCCATGCAAATTCTGGTAGGAGTCCTACAGACTGTGCCCAAAGTAGCAAGAGTGTCAAGGAAGCATGGACTACAACAGAGCAGCTCCAGTTTACTATTTTTGCTGCTCATGGAATTTCAAGTAATTGGGTATCAAATTATGAAAAATACTACTTGATATGTTCACTGTCTCACAATGGAAAGGATCTTTTTAAACCTATTCAATCAAAGAAAGTCGGTACGTATAAAAACTTCTTCTATCTTATTAAATGGGATGAACTAATCATTTTTCCTATCCAGATATCACAATTGCCATTAGAATCAGTTCTTCACCTTACTCTTTTTGGAATTTTAAATCAGAGCAGTGGAAGTTCCCCTGATTCTAATAAGCAGAGAAAGGGACCAGAAGCTTTGGGCAAAGTTTCTTTACCTCTTTTTGACTTTAAACGGTTTTTAACATGTGGAACTAAACTTCTATATCTTTGGACTTCATCACATACAAATTCTGTTCCTGGAACAGTTACCAAAAAAGGATATGTCATGGAAAGAATAGTGCTACAGGTTGATTTTCCTTCTCCTGCATTTGATATTATTTATACAACTCCTCAAGTTGACAGAAGCATTATACAGCAACATAACTTAGAAACACTAGAGAATGATATAAAAGGGAAACTTCTTGATATTCTTCATAAAGACTCATCACTTGGACTTTCTAAAGAAGATAAAGCTTTTTTATGGGAGAAACGTTATTATTGCTTCAAACACCCAAATTGTCTTCCTAAAATATTAGCAAGCGCCCCAAACTGGAAATGGGTTAATCTTGCCAAAACTTACTCATTGCTTCACCAGTGGCCTGCATTGTACCCACTAATTGCATTGGAACTTCTTGATTCAAAATTTGCTGATCAGGAAGTAAGATCCCTAGCTGTGACCTGGATTGAGGCCATTAGTGATGATGAGCTAACAGATCTTCTTCCACAGTTTGTACAAGCTTTGAAATATGAAATTTACTTGAATAGTTCATTAGTGCAATTCCTTTTGTCCAGGGCATTGGGAAATATCCAGATAGCACACAATTTATATTGGCTTCTCAAAGATGCCCTGCATGATGTACAGTTTAGTACCCGATACGAACATGTTTTGGGTGCTCTCCTGTCAGTAGGAGGAAAACGACTTAGAGAAGAACTTCTAAAACAGACGAAACTTGTACAGCTTTTAGGAGGAGTAGCAGAAAAAGTAAGGCAGGCTAGTGGATCAGCCAGACAGGTTGTTCTCCAAAGAAGTATGGAACGAGTACAGTCCTTTTTTCAGAAAAATAAATGCCGTCTCCCTCTCAAGCCAAGTCTAGTGGCAAAAGAATTAAATATTAAGTCGTGTTCCTTCTTCAGTTCTAATGCTGTCCCCCTAAAAGTCACAATGGTGAATGCTGACCCTATGGGAGAAGAAATTAATGTCATGTTTAAGGTTGGTGAAGATCTTCGGCAAGATATGTTAGCTTTACAGATGATAAAGATTATGGATAAGATCTGGCTTAAAGAAGGACTAGATCTGAGGATGGTAATTTTCAAATGTCTCTCAACTGGCAGAGATCGAGGCATGGTGGAGCTGGTTCCTGCTTCCGATACCCTCAGGAAAATCCAAGTGGAATATGGTGTGACAGGATCCTTTAAAGATAAACCACTTGCAGAGTGGCTAAGGAAATACAATCCCTCTGAAGAAGAATATGAAAAGGCTTCAGAGAACTTTATCTATTCCTGTGCTGGATGCTGTGTAGCCACCTATGTTTTAGGCATCTGTGATCGACACAATGACAATATAATGCTTCGAAGCACGGGACACATGTTTCACATTGACTTTGGAAAGTTTTTGGGACATGCACAGATGTTTGGCAGCTTCAAAAGGGATCGGGCTCCTTTTGTGCTGACCTCTGATATGGCATATGTCATTAATGGGGGTGAAAAGCCCACCATTCGTTTTCAGTTGTTTGTGGACCTCTGCTGTCAGGCCTACAACTTGATAAGAAAGCAGACAAACCTTTTTCTTAACCTCCTTTCACTGATGATTCCTTCAGGGTTACCAGAACTTACAAGTATTCAAGATTTGAAATACGTTAGAGATGCACTTCAACCCCAAACTACAGACGCAGAAGCTACAATTTTCTTTACTAGGCTTATTGAATCAAGTTTGGGAAGCATTGCCACAAAGTTTAACTTCTTCATTCACAACCTTGCTCAGCTTCGTTTTTCTGGTCTTCCTTCTAATGATGAGCCCATCCTTTCATTTTCACCTAAAACATACTCCTTTAGACAAGATGGTCGAATCAAGGAAGTCTCTGTTTTTACATATCATAAGAAATACAACCCAGATAAACATTATATTTATGTAGTCCGAATTTTGAGGGAAGGACAGATTGAACCATCATTTGTCTTCCGAACATTTGACGAATTTCAGGAACTTCACAATAAGCTCAGTATTATTTTTCCACTTTGGAAGTTACCAGGCTTTCCTAATAGGATGGTTCTAGGAAGAACACACATAAAAGATGTAGCAGCCAAAAGGAAAATTGAGTTAAACAGTTACTTACAGAGTTTGATGAATGCTTCAACGGATGTAGCAGAGTGTGATCTTGTTTGTACTTTCTTCCACCCTTTACTTCGTGATGAGAAAGCTGAAGGGATAGCTAGGTCTGCAGATGCAGGTTCCTTCAGTCCTACTCCAGGCCAAATAGGAGGAGCTGTGAAATTATCCATCTCTTACCGAAATGGTACTCTTTTCATCATGGTGATGCATATCAAAGATCTTGTTACTGAAGATGGAGCTGACCCAAATCCATATGTCAAAACATACCTACTTCCAGATAACCACAAAACATCCAAACGTAAAACCAAAATTTCACGAAAAACGAGGAATCCGACATTCAATGAAATGCTTGTATACAGTGGATATAGCAAAGAAACCCTAAGACAGCGAGAACTTCAACTAAGTGTACTCAGTGCAGAATCTCTGCGGGAGAATTTTTTCTTGGGTGGAGTAACCCTGCCTTTGAAAGATTTCAACTTGAGCAAAGAGACGGTTAAATGGTATCAGCTGACTGCGGCAACATACTTGTAA |
